# The Influence of Molecular Reach and Diffusivity on the Efficacy of Membrane-Confined Reactions

**DOI:** 10.1016/j.bpj.2019.08.023

**Published:** 2019-08-28

**Authors:** Ying Zhang, Lara Clemens, Jesse Goyette, Jun Allard, Omer Dushek, Samuel A. Isaacson

**Affiliations:** 1 Department of Mathematics and Statistics, Boston University, Boston, Massachusetts; 2 Center for Complex Biological Systems, University of California-Irvine, Irvine, California; 3 School of Medical Sciences, University of New South Wales, Sydney, Australia; 4 Sir William Dunn School of Pathology, University of Oxford, Oxford, United Kingdom

## Abstract

Signaling by surface receptors often relies on tethered reactions whereby an enzyme bound to the cytoplasmic tail of a receptor catalyzes reactions on substrates within reach. The overall length and stiffness of the receptor tail, the enzyme, and the substrate determine a biophysical parameter termed the molecular reach of the reaction. This parameter determines the probability that the receptor-tethered enzyme will contact the substrate in the volume proximal to the membrane when separated by different distances within the membrane plane. In this work, we develop particle-based stochastic reaction-diffusion models to study the interplay between molecular reach and diffusion. We find that increasing the molecular reach can increase reaction efficacy for slowly diffusing receptors, whereas for rapidly diffusing receptors, increasing molecular reach reduces reaction efficacy. In contrast, if reactions are forced to take place within the two-dimensional plasma membrane instead of the three-dimensional volume proximal to it or if molecules diffuse in three dimensions, increasing molecular reach increases reaction efficacy for all diffusivities. We show results in the context of immune checkpoint receptors (PD-1 dephosphorylating CD28), a standard opposing kinase-phosphatase reaction, and a minimal two-particle model. The work highlights the importance of the three-dimensional nature of many two-dimensional membrane-confined interactions, illustrating a role for molecular reach in controlling biochemical reactions.

## Significance

Signaling by surface receptors often relies on tethered reactions wherein enzyme binding to a receptor’s cytoplasmic tail catalyzes reactions with nearby substrates. The length and stiffness of the tail, enzyme, and substrate can be summarized by the molecular reach of the reaction. The role of molecular reach in modulating the efficacy of signaling reactions is poorly understood. We show that increasing reach increases reaction efficacy when receptor diffusion is slow but decreases reaction efficacy when diffusion is fast. This switch in efficacy results from the tails of membrane-confined molecules being able to explore the three-dimensional volume proximal to the membrane. The work highlights the three-dimensional nature of two-dimensional membrane interactions, identifying reach as a control parameter for reaction efficacy.

## Introduction

The ability of cells to sense their extracellular environment and make decisions relies on a diverse set of biochemical signaling reactions. Common to many of these reactions is the binding or tethering of an enzyme near its substrate before catalysis. Tethered signaling reactions are therefore controlled not only by binding affinities and catalytic specificities but also by the properties of tethers that control the molecular reach of the reaction (defined below). Examples of tethered signaling reactions include those that take place on scaffolds (1, 2) and those that take place on the cytoplasmic tails of cell surface receptors. Tethering has also been used in synthetic biology to modulate endogenous signaling pathways (3, 4). Although binding and catalytic reactions have been extensively studied experimentally and theoretically, the role of molecular reach is less well-understood.

**Figure 1:**
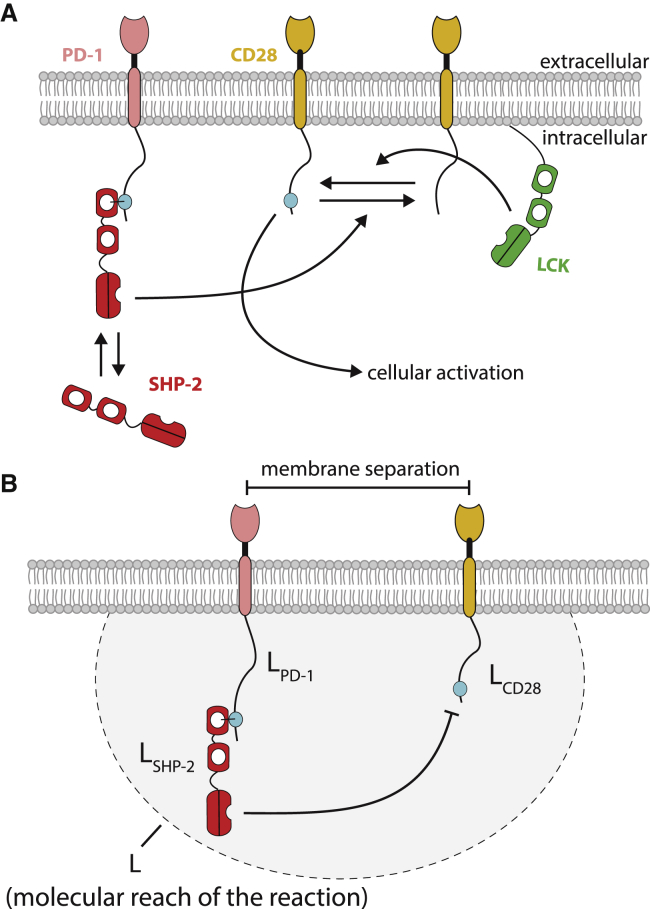
Illustration of tethered signaling reactions regulating the phosphorylation of the costimulatory surface receptor CD28 expressed on T cells. (*A*) The membrane-anchored tyrosine kinase LCK is known to phosphorylate CD28. The cytoplasmic tyrosine phosphatase SHP-2 is known to dephosphorylate CD28 when tethered (or bound) to the cytoplasmic tail of the inhibitory receptor PD-1. The kinase (LCK) and both receptors (CD28, PD-1) diffuse within the 2D membrane plane. (*B*) The rate of CD28 dephosphorylation by SHP-2 will be controlled, in part, by the molecular reach of the reaction (*L*), with a larger reach generally increasing reaction rates when molecules are further apart. The molecular reach of the reaction will depend on the molecular reach of the individual components (*L*_PD-1_, *L*_SHP-2_, and *L*_CD28_). We estimate the molecular reach for this reaction to be *L* ≈ 8.5 nm (see Materials and Methods). To see this figure in color, go online.

In the case of noncatalytic tyrosine-phosphorylated receptors (NTRs) (5), cytosolic enzymes first bind to their unstructured cytoplasmic tails before catalyzing reactions within reach. As a specific example, we consider the regulation of the NTR group member CD28 (Fig. 1 *A*). This costimulatory receptor is expressed on T cells of the adaptive immune system and is known to initiate signals important for their activation (6). Phosphorylation of CD28 is mediated by the membrane-anchored SRC-family kinase LCK. It has been shown recently that CD28’s dephosphorylation is mediated by the NTR group member programmed cell death protein 1 (PD-1) (7, 8). This inhibitory receptor contains a tyrosine motif (ITSM) that serves as a docking site for the SH2 domain of the cytoplasmic tyrosine phosphatase SHP-2. When tethered to PD-1, SHP-2 is able to dephosphorylate tyrosines within reach, including those on CD28. Therefore, in addition to diffusion of these receptors within the membrane plane, it is expected that the tether will also play a role in controlling the ability of PD-1 to inhibit T-cell activation.

In this example, the rate of CD28 dephosphorylation is expected to be influenced by the molecular reach of the reaction (Fig. 1 *B*). Molecular reach determines the probability that the enzyme will contact the substrate when the two receptors are at a defined separation distance on the membrane. The overall molecular reach of the reaction is determined by the reach of the cytoplasmic tail of PD-1 (*L*_PD-1_), the reach of SHP-2 (*L*_SHP-2_), and the reach of the cytoplasmic tail of CD28 (*L*_CD28_). Here, *L*_PD-1_, *L*_SHP-2_, and *L*_CD28_ will, in turn, depend on the respective length and stiffness properties of each component. By using the worm-like chain (WLC) polymer model, the overall molecular reach of the reaction can be defined as the square root of the squared sum of the individual reach parameters: $L=\sqrt{L_{\text{PD-1}}^{2}+L_{\text{SHP-2}}^{2}+L_{\text{CD}28}^{2}}$. Experimental estimates of the molecular reach have yet to be reported, but we estimate the molecular reach for this reaction to be approximately *L* = 8.5 nm (see Materials and Methods for further details). We note that binding, catalysis, and molecular reach as defined in these reactions are structurally independent, and therefore, changes to molecular reach are not expected to alter the catalytic or binding rate constants.

We note that SHP-2 and the homologous phosphatase SHP-1 are recruited to a variety of different receptors and act on a diverse set of substrates (9). It follows that the molecular reach for SHP-2 (or SHP-1) catalyzing a reaction on any given substrate (from different receptors) or from any given receptor (to different substrates) may exhibit wide variations. Indeed, the cytoplasmic tails of NTRs vary in their overall length (10).

To understand the role of molecular reach and diffusion in tethered signaling, we developed a particle-based convergent reaction-diffusion master equation (CRDME) model for the reaction and diffusion of individual receptors, kinases and phosphatases (11, 12). Importantly, when simulating reactions between molecules confined to the two-dimensional (2D) plasma membrane, we explicitly allowed their tails to explore the three-dimensional (3D) volume proximal to the membrane by using a physiological 3D kernel that depends on the molecular reach (Fig. 1 *B*). This model builds on our previous work investigating tethered reactions without diffusion in surface plasmon resonance assays (10).

Using our particle model, we first study the dephosphorylation of CD28 by PD-1 as the molecular reach of the reaction is varied. We find that the potency of PD-1 increases as the molecular reach increases for slowly diffusing receptors. In contrast, for rapidly diffusing receptors, we find that increases in molecular reach reduce PD-1 potency. We show that this switch in potency as the molecular reach increases also holds in a commonly used biochemical model of reversible phosphorylation by kinases and phosphatases. In both biochemical models, we find that the switch is lost if membrane reactions are modeled using an idealized kernel that forces reactions within the 2D membrane plane. Using a simplified two-particle model that can be solved analytically, we reproduce these results. We then show that the switch arises from the constraint imposed by the molecules diffusing within the plasma membrane, which prevents the tethers from reaching all possible configurations in which a reactive encounter could occur. Consistent with this, the switch is lost if molecules continue to interact using the 3D physiological kernel but are instead allowed to diffuse in 3D. In this case, the region where the molecules diffuse allows the tethers to sample all possible configurations in which a reactive encounter can occur. Our work highlights the 3D nature of 2D membrane-confined reactions and suggests a possible unexpected role for molecular reach in controlling biochemical reactions.

## Materials and Methods

### CRDME SSA simulations

With the exception of our final simplified model, in which only one molecule diffuses, we study each of the biological models by Monte Carlo simulation of particle-based stochastic reaction-diffusion systems. Our simulation method is the CRDME stochastic simulation algorithm (SSA) (11, 12). Here, the diffusion of individual molecules is approximated by a continuous time random walk of the molecules hopping between voxels of a Cartesian mesh. First-order reactions occur with an exponential clock, sampled independently for each possible first-order reaction. Bimolecular reactions between two molecules occur with a separation dependent probability per time (derived from the Gaussian kernel *k*_cat_*σ*(*r*;*L*) for separation *r*, catalytic rate *k*_cat_, and molecular reach *L*; see the next section and (12)). In this way, we approximate the diffusion and reactions of the molecules by a jump process. Note that unlike the lattice reaction-diffusion master equation model (13, 14), the CRDME converges to an appropriate spatially continuous particle reaction-diffusion model, the volume-reactivity model, as the lattice spacing is taken to zero. In Supporting Materials and Methods, Section S1, we provide a more detailed description of how the CRDME is formulated.

**Figure 2:**
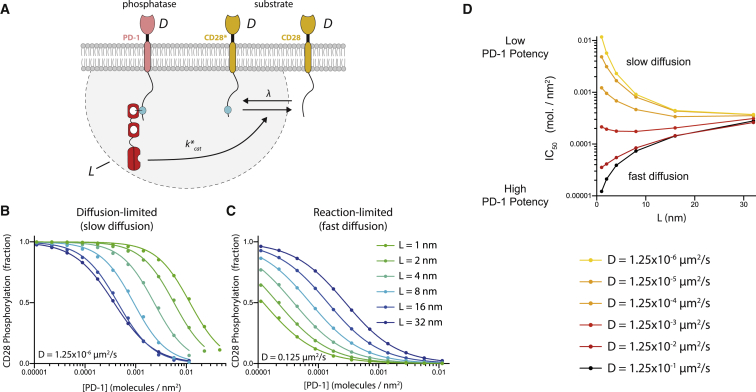
The potency of PD-1 receptor can increase or decrease as the molecular reach of the reaction increases depending on diffusivity. (*A*) A schematic of species and biochemical reactions in our stochastic spatial model. (*B* and *C*) Steady-state fraction of phosphorylated CD28 ([CD28^∗^]/([CD28^∗^] + [CD28])) versus [PD-1] for different values of the molecular reach for (*B*) a smaller diffusion coefficient and (*C*) a larger diffusion coefficient. (*D*) Concentration of [PD-1] producing a 50% reduction in CD28 phosphorylation (also known as IC_50_ or potency) over the molecular reach of the reaction for different values of the diffusion coefficient. Note that a large potency equates to a small value of IC_50_. Parameters are summarized in Table 1. To see this figure in color, go online.

**Figure 3:**
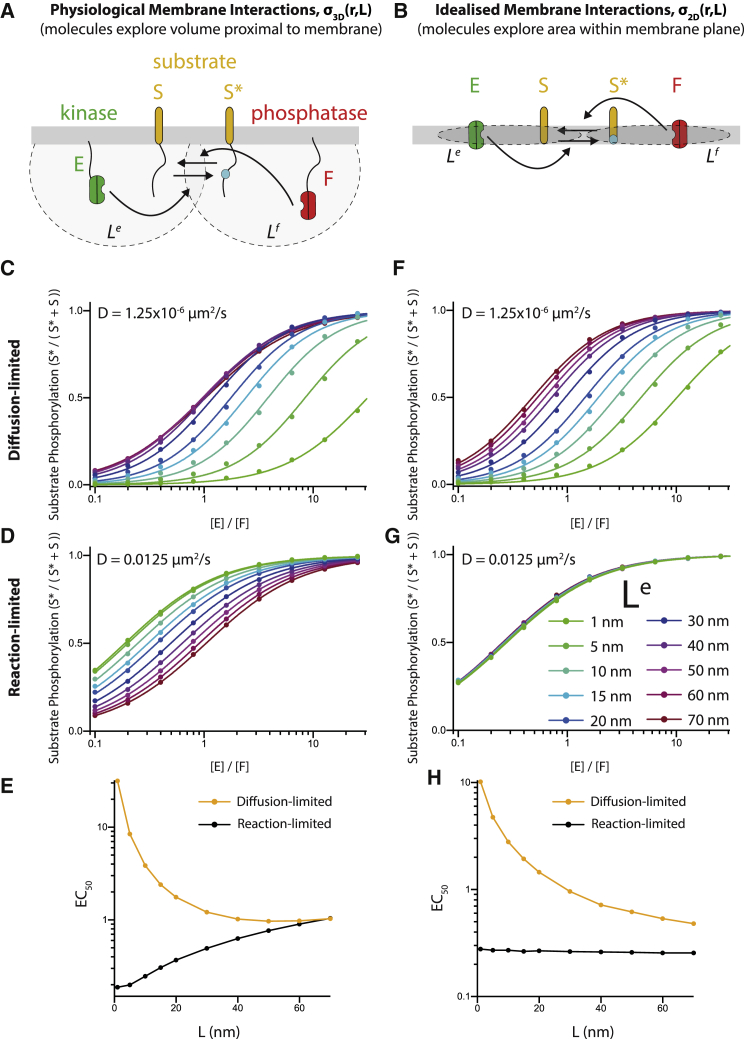
The switch in efficacy when increasing the molecular reach is only observed when explicitly allowing enzymes to explore the volume proximal to the membrane. Here, *L*^*e*^ is varied from 1 to 70 nm to explore the effect of increasing reach. (*A* and *B*) A schematic of the biochemical model showing the reversible modification of a substrate by a kinase and phosphatase with reactions taking place (*A*) within a volume proximal to the membrane or (*B*) artificially confined to the plane of the membrane. The phosphorylation of the substrate is calculated in the steady state for the physiological geometry (*C* and *D*) or the idealized geometry (*F* and *G*) when diffusion is limiting reactions (*C* and *F*) or when it is not limiting (*D* and *G*). Calculations are shown for different values of the molecular reach parameter for the kinase (legend in *G* applies to *C*, *D*, *F*, and *G*). The potency of the kinase over the molecular reach is shown for the (*E*) physiological and (*H*) idealized geometry. All parameters are summarized in Table 2. Note that when using the 2D kernel, *σ*_2D_, the two-dimensional catalytic rates were given by $k_{\text{cat}}^{e}=\left(4/3\right)$ × 10^5^*μ*M^−1^ s^−1^ m^−1^ = 221.3736 (nm)^2^ s^−1^ and $k_{\text{cat}}^{f}=\left(1/3\right)$ × 10^5^*μ*M^−1^ s^−1^ m^−1^ = 55.3434 (nm)^2^ s^−1^. To see this figure in color, go online.

To study the first two models in the Results and the simplified two-particle model (Eq. S13), we used the CRDME SSA on a square (or cubic) domain with sides of length 300 nm. The first two models had periodic boundary conditions on the sides of the square (cube), whereas the simplified two-particle model used a reflecting Neumann boundary condition; see Eq. S13. The domain was discretized into a Cartesian mesh of 2^16^ square voxels in 2D and 2^24^ cubic voxels in 3D. Each curve in Figs. 2, *B–D*, 3, *C–H*, and S4 was estimated from 50,000 simulations using the parameters in Tables 1 and 2. For the first two models, simulations were run until individual trajectories reached steady state. Our protocol for determining when steady state was reached is described in Supporting Materials and Methods, Section S8. For the simplified model (Eq. S13), simulations were run until the two molecules reacted, with the corresponding reaction time then saved. As shown in Supporting Materials and Methods, Section S7, the qualitative dependence of the models on the diffusivity of molecules and the molecular reach of reactions was found to be relatively insensitive to the domain size (for molecular reaches much smaller than the domain width).

**Table 1:** Parameters for the PD-1 Model

Parameter	Description	Value
*D*	diffusion coefficient	indicated *μ*m^2^ s^−1^
[PD-1]	PD-1 concentration	indicated nm^−2^
[CD28]	CD28 concentration	0.0001 nm^−2^
*λ*	phosphorylation rate	1.0 s^−1^
$k_{\text{cat}}^{*}$	catalytic efficiency	0.1 *μ*M^−1^ s^−1^
*L*	molecular reach	indicated nm
Domain	periodic square	300 nm × 300 nm

**Table 2:** Parameters for Reversible Phosphorylation Model

Parameter	Description	Value
[S]	substrate concentration	100 *μ*m^−2^
[E]	kinase concentration	indicated
[F]	phosphatase concentration	112 *μ*m^−2^
*D*^*S*^	substrate diffusivity	indicated *μ*m^2^ s^−1^
*D*^*e*^	kinase diffusivity	indicated *μ*m^2^ s^−1^
*D*^*f*^	phosphatase diffusivity	6.25 × 10^−4^*μ*m^2^ s^−1^
$k_{\text{cat}}^{e}$	kinase catalytic efficiency	0.04 *μ*M^−1^ s^−1^
$k_{\text{cat}}^{f}$	phosphatase catalytic efficiency	0.01 *μ*M^−1^ s^−1^
*L*^*e*^	kinase molecular reach	indicated nm
*L*^*f*^	phosphatase molecular reach	15 nm
Domain	periodic square	300 nm × 300 nm

### Derivation of probability density kernel *σ*

In our CRDME-based models, the kernel *σ*_3D_ determines the probability density that an individual tethered substrate (e.g., the phosphorylation site on the cytoplasmic tail of CD28) will come in contact with an individual tethered enzyme (e.g., the catalytic pocket of the phosphatase domain of SHP-2 tethered to the cytoplasmic tail of PD-1) at different separation distances between the membrane tether positions. That is, if the substrate’s tether is at position ***x*** in the membrane and the enzyme’s tether is a position ***y*** in the membrane, the separation distance between the tether positions is *r* = |***x*** − ***y***|. By assuming that the substrate and enzyme can be approximated by the WLC polymer model, an analytical expression for the probability density kernel can be obtained (10, 15):

(1) $$\sigma_{3\text{D}}\left(r;L\right)={\left(\frac{3}{2\pi L^{2}}\right)}^{3/2}\text{exp}\left(-\frac{3r^{2}}{2L^{2}}\right),$$

where *L* is the molecular reach for the reaction and is given by the square root of the squared sum of the molecular reach of individual reaction components (10). In the specific example of PD-1 dephosphorylating CD28 (Figs. 1 and 2), $L=\sqrt{L_{\text{PD-1}}^{2}+L_{\text{SHP-1}/2}^{2}+L_{\text{CD}28}^{2}}$. For the reversible phosphorylation model that we consider in the Results (see Fig. 3),

$$\text{S}+\text{E}\overset{k_{\text{cat}}^{e}\sigma \left(r;L^{e}\right)}{⇀}{\text{S}}^{*}+\text{E,}$$

$${\text{S}}^{*}+\text{F}\overset{k_{\text{cat}}^{f}\sigma \left(r;L^{f}\right)}{⇀}\text{S}+\text{F,}$$

the molecular reach for the first (kinase) reaction would be $L^{e}=\sqrt{L_{\text{E}}^{2}+L_{\text{S}}^{2}}$ and for the second (phosphatase) reaction $L^{f}=\sqrt{L_{\text{F}}^{2}+L_{\text{S}}^{2}}$. Here, *L*_E_ is the molecular reach of the kinase E, *L*_F_ is the molecular reach of the phosphatase F, and *L*_S_ is the molecular reach of the substrate S and phosphorylated substrate S^∗^.

We note that the original derivation of Eq. 1 assumed that tethers explored free space instead of the half-space imposed by the plasma membrane (10, 15). Simulations revealed that the free-space kernel agrees with the half-space kernel up to a scaling constant (see Supporting Materials and Methods, Section S10), and therefore, to keep the models in the main text computationally efficient, we have used Eq. 1.

In a number of simulations, we replace *σ*_3D_ by an idealized 2D interaction kernel to artificially force molecules to interact within the plane of the membrane, given by

(2) $$\sigma_{2\text{D}}\left(r;L\right)=\frac{3}{2\pi L^{2}}\text{exp}\left(-\frac{3r^{2}}{2L^{2}}\right).$$

A plot of *σ*_3D_ and *σ*_2D_ is provided in Fig. S1. *σ*_2D_ arises by restricting the physical diffusion of the tails to the membrane plane. In simulations using *σ*_2D_, we still assume that reactions between catalytic sites that are in contact, characterized by catalytic rate *k*_cat_, is a distinct process from the probability the sites are in contact (which is determined by *σ*_2D_). As such, we still assume that *k*_cat_ is structurally independent of the reach.

### Estimating the molecular reach *L* for PD-1 dephosphorylating CD28

In the absence of experimental measurements, we approximate the molecular reach parameter for PD-1 dephosphorylating CD28. The molecular reach parameter for CD28 is simply $L_{\text{CD}28}=\sqrt{l_{p}l_{c}}$, where *l*_*p*_ is the persistence length and *l*_*c*_ is the contour length. The persistence length for unstructured amino acid chains has previously been estimated to be *l*_*p*_ = 0.4 nm (15, 16). The contour length of CD28 can be estimated using *l*_*c*_ = (0.4 nm) × *N*, where *N* is the number of amino acids between the membrane and the phosphorylated tyrosine and 0.4 nm is the C_*α*_-C_*α*_ bond length in a polypeptide chain. The key activator tyrosine in CD28 is the YMNM motif located 11 amino acids from the membrane, and therefore, *L*_CD28_ = 1.3 nm. Similarly, the molecular reach parameter for PD-1 is estimated to be *L*_PD-1_ = 3.0 nm, where the number of amino acids between the membrane and the tyrosine in the ITSM that binds SHP-2 is *N* = 56.

The molecular reach of the enzyme SHP-2 is more difficult to estimate because it is composed of three structured domains with flexible linkers: N-SH2(linker)C-SH2(linker)protein tyrosine phosphatase (Fig. 1). Given that SHP-2 docks to its substrate primarily using the N-SH2 and catalyzes reactions with its protein tyrosine phosphatase catalytic domain, an upper bound for the molecular reach can be estimated by adding up the distances of the structured domains and the peptide linkers to obtain a maximal reach of 17.1 nm. However, the flexible linkers are unlikely to be maximally stretched, and therefore, a more realistic estimate is obtained by assuming a persistence length of 0.4 nm for the linkers that leads to an overall reach of 7.9 nm for SHP-2. We note that estimating the reach directly from the crystal structure (Protein Data Bank [PDB]: 2SHP) produces a value of 3.6 nm, but this value is for a single conformation of SHP-2.

In summary, the molecular reach of the reaction for SHP-2 bound PD-1 dephosphorylating CD28 can be approximated to be *L* = 8.5 nm.

## Results

### A larger molecular reach can increase or decrease PD-1 receptor potency depending on diffusion

To investigate the influence of molecular reach on the ability of PD-1 to inhibit CD28, we developed a CRDME particle model (see Materials and Methods). The model included unphosphorylated CD28, phosphorylated CD28, and PD-1 bound to SHP-2, with all molecules able to diffuse in the plasma membrane (Fig. 2 *A*). We explicitly included the effects of molecular reach by modeling the dephosphorylation of CD28 by PD-1 as a second-order reaction whose rate was dependent on the separation distance between the molecules within the membrane (*r*),

(3) $${\text{CD}28}^{*}+\text{PD-1}\overset{k_{\text{cat}}^{*}\sigma_{\text{3D}}\left(r;L\right)}{⇀}\text{CD}28+\text{PD-1},$$

where $k_{\text{cat}}^{*}$ is the catalytic efficiency and *L* is the molecular reach of the reaction. The function *σ*_3D_ is the probability density (in units of molecules/nm^3^ or *μ*M) for finding the enzyme and substrate at the same location when their respective receptors are separated by a distance *r* within the plane of the plasma membrane. It depends only on the membrane position of the receptors but accounts for the diffusive motion of the tethered enzyme and substrate within the cytosol; see Materials and Methods. We calculate *σ*_3D_ under the assumption that PD-1 and CD28 can be approximated by the WLC polymer model, obtaining the Gaussian interaction given by Eq. 1 (see Materials and Methods).

We focus on the effects of molecular reach for the dephosphorylation reactions, and therefore, we have introduced two simplifications to the model. First, we do not explicitly include the recruitment of SHP-2 to PD-1. Second, we do not explicitly model LCK molecules but instead model CD28 phosphorylation by a first-order reaction ($\text{CD}28\overset{\lambda }{⇀}\text{CD}28^{*}$). These simplifications, which decrease the computational complexity of the model by reducing the number of molecules that must be resolved in simulations, are not expected to alter our conclusions: first, explicitly modeling SHP-2 recruitment would reduce the effective concentration of PD-1 bound to SHP-2 that can act on CD28 and therefore would be expected to act as a correction factor for the concentration of PD-1. Second, explicitly modeling LCK would not be expected to alter any conclusions because parameters associated with it were not varied.

As output of the model, we calculated the steady-state fraction of phosphorylated CD28 as the concentration of PD-1 was increased. We first focused on a situation in which diffusion is minimal, which may be the case when immune receptors bind their ligands (17, 18), interact with the cytoskeleton (19, 20), and/or cluster (21). As expected, increasing the concentration of PD-1 reduced phosphorylation of CD28 (Fig. 2 *B*). In this case, we found that increasing the molecular reach of the reaction increased the potency of PD-1 so that fewer PD-1 molecules were necessary to achieve the same level of inhibition. Unexpectedly, when using a diffusion coefficient representative of free mobility on the plasma membrane for transmembrane receptors (19, 22), we found that increasing the molecular reach decreased the potency of PD-1 so that more PD-1 molecules were necessary to achieve the same level of inhibition (Fig. 2 *C*).

We quantified the potency of PD-1 by calculating the concentration of PD-1 required to reduce the phosphorylation of CD28 by 50% (also known as IC_50_). A plot of IC_50_ over *L* shows that PD-1 potency increases for small but decreases for large diffusion coefficients, with a transition at intermediate values of the diffusion coefficient (*D* = 0.00125 *μ*m^2^/s) at which potency is largely unchanged (Fig. 2 *D*). Taken together, we find a switch in the effect of changing molecular reach, with larger reaches increasing receptor potency when diffusion is slow but decreasing receptor potency when diffusion is fast.

### Effect of molecular reach in physiological and idealized membrane reactions

A key novelty of our membrane-bound protein reaction model is in accounting for reactions involving sites on molecular tails, which move through the volume proximal to the membrane. This is achieved through the use of the 3D interaction kernel *σ*_3D_, which accounts for the motion and stiffness properties of the tails, bound enzymes, and substrates (see Materials and Methods). To determine the importance of the 3D kernel to the observed switch in reaction efficacy, we replaced the physiological kernel with an idealized 2D interaction kernel *σ*_2D_ (see Eq. 2). This 2D kernel forced chemical interactions to only occur within the plane of the membrane (see Fig. S1), as in previous models (23). To simulate this and to generalize beyond the specific example of PD-1 acting on CD28, we reformulated the biochemistry of the model to a widely used scheme for the reversible modification of a substrate by a kinase and phosphatase (Fig. 3, *A* and *B*; (23, 24, 25)),

$$\text{S}+\text{E}\overset{k_{\text{cat}}^{e}\sigma \left(r;L^{e}\right)}{⇀}{\text{S}}^{*}+\text{E,}$$

$${\text{S}}^{*}+\text{F}\overset{k_{\text{cat}}^{f}\sigma \left(r;L^{f}\right)}{⇀}\text{S}+\text{F,}$$

where S, E, and F are the substrate, kinase, and phosphatase, respectively, and ^∗^ indicates the phosphorylation modification (Fig. 3, *A* and *B*). As before, we allowed for diffusion of all chemical species and highlight that the rate of these enzymatic reactions is proportional to the catalytic efficacies ($k_{\text{cat}}^{e}$ and $k_{\text{cat}}^{f}$) multiplied by the probability densities (*σ*(*r*;*L*^*e*^) and *σ*(*r*;*L*^*f*^) for physiological 3D or idealized 2D interactions). The latter explicitly depends on the separation distance between the molecules in the simulation (*r*) and on the reaction molecular reach: *L*^*e*^ for the kinase phosphorylating the substrate and *L*^*f*^ for the phosphatase dephosphorylating the substrate.

We calculated the steady-state fraction of phosphorylated substrate as the number of kinase molecules was increased. Using the physiological 3D kernel, we reproduced the results for PD-1 (Fig. 2), in which increasing the molecular reach increased the potency of the kinase when diffusion was slower but decreased its potency when diffusion was faster (Fig. 3, *C* and *D*). When using the idealized 2D kernel, we found that increasing the molecular reach of the reaction increased the potency of the kinase when diffusion was slower (Fig. 3 *F*), but when diffusion was faster, it had no effect on the potency of the kinase (Fig. 3 *G*). As before, we summarized these results by calculating the potency of the kinase as a function of the molecular reach for the physiological and idealized kernels (Fig. 3, *E* and *H*). We confirmed that using the idealized 2D kernel in the PD-1 model of the last section also led the molecular reach to have a minimal effect in the reaction-limited, i.e., fast diffusion, regime (Fig. S2).

Taken together, these results highlight that the switching behavior in potency as molecular reach is increased is observed when using a physiological 3D kernel but not an idealized 2D kernel. We conclude that the 3D nature of 2D interactions can have profound effects on biochemical reaction rates.

### A minimal two-particle Doi model explains molecular reach phenotype

The preceding models demonstrate a clear switch in how the efficacy (quantified as potency) of tethered signaling reactions depends on molecular reach for large versus small diffusivities when molecules are confined to the 2D plasma membrane. They also suggest that such a switch may not be possible when molecules are forced to interact within the plane of the membrane. To understand what gives rise to this switch and why it is not present when the molecules react in the membrane plane, we developed a simplified two-particle Doi model that could be solved analytically.

**Figure 4:**
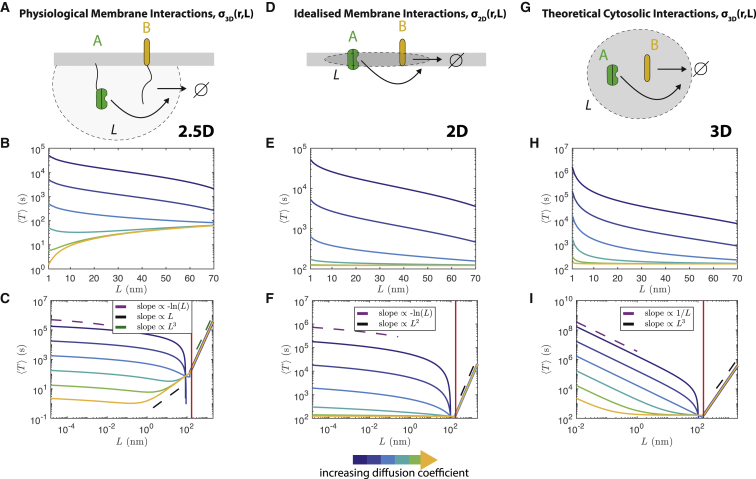
The well-mixed mean reaction time (MRT), 〈T〉, only demonstrates a switch in dependence on molecular reach for small versus large diffusivities when considering membrane-bound molecules with cytosolic tails that react in 3D (2.5D model). (*A*), (*D*) and (*G*) illustrate the effective 2.5D, 2D, and 3D model regions in which the proteins (*darker region*) and their tails (*region with dashed border*) can diffuse. In all graphs, solid lines correspond to the asymptotic expansions in Eq. 11a (*B* and *C*), Eq. 11b (*E* and *F*), or Eq. 11c (*H* and *I*). Dashed lines give scaling behavior as a function of *L*. (*B*) 2.5D model well-mixed MRT over physical parameter range. (*C*) Same as (*B*) but showing an expanded range of *L*-values. (*E*) 2D model well-mixed MRT over physical parameter range. (*F*) Same as (*E*) but showing an expanded range of *L*-values. (*H*) 3D model well-mixed MRT over physical parameter range. (*I*) Same as (*H*) but showing an expanded range of *L*-values. In (*C*), (*F*), and (*I*), an extreme range of *L*-values is used to demonstrate the different scaling regimes of 〈T〉 in *L*. The vertical red line gives the *L*-value such that *ε*/*R* = 1, corresponding to when the Doi interaction distance, *ε*, is equal to the domain radius, *R*. Note that as *ε* → *R* from below, the asymptotic expansions break down because ε/R 1. For (*B*), (*C*), (*H*), and (*I*), the catalytic rate *k*_cat_ is 0.1 *μ*M^−1^ s^−1^. For (*E*) and (*F*), the 2D catalytic rate *k*_cat_ is (1/3) × 10^6^*μ*M^−1^ s^−1^ m^−1^ = 553.4341 (nm)^2^ s^−1^. Diffusion coefficients (*blue* to *yellow*): 1.25 × 10^−6^, 1.25 × 10^−5^, 1.25 × 10^−4^, 1.25 × 10^−3^, 1.25 × 10^−2^, 0.1 *μ*m^2^ s^−1^. To see this figure in color, go online.

We consider a system containing just one A molecule and one B molecule, which can undergo the annihilation reaction

$$\text{A}+\text{B}\overset{k_{\text{cat}}\sigma \left(r;L\right)}{⇀}\emptyset ,$$

and assume that the A molecule is stationary and located at the origin, whereas the B molecule diffuses. We will consider three cases: the physiological model in which the B molecule diffuses in 2D and tails interact in 3D (through the 3D Gaussian, *σ*_3D_(*r*;*L*)), a model in which the B molecule diffuses in 2D but tails are forced to only interact in 2D (through the 2D Gaussian, *σ*_2D_(*r*;*L*)), and a model in which the B molecule diffuses in 3D and tails interact in 3D (through the 3D Gaussian, *σ*_3D_(*r*;*L*)). In the remainder, we denote these three combinations as the 2.5D, 2D, and 3D models, respectively (Fig. 4).

In the Doi model, we assume the B molecule diffuses with diffusivity *D* within a circle (sphere) of radius *R* about the origin. *R* was chosen so that the area (volume) of the circle (sphere) was identical to that of the square (cube) with sides of length 300 nm used in the preceding sections. We replace the Gaussian interaction *k*_cat_*σ*(*r*;*L*) by an approximating indicator function *λ*1_[0,*ε*]_(*r*), defined by

$$\lambda 1_{\left[0,\varepsilon \right]}\left(r\right)=\{\begin{array}{cc} \lambda , & 0\leq r\leq \varepsilon ,\\ 0, & \varepsilon <r. \end{array}$$

Here, *λ* corresponds to the probability per time the molecules react when within a reaction-radius, *ε*, of each other.

The mean reaction time (MRT) *w*(*r*) for a diffusing molecule that is initially placed a distance *r* from the origin then satisfies

(4) $$\begin{array}{l} \frac{D}{r^{d-1}}\frac{d}{dr}\left(r^{d-1}\frac{dw}{dr}\left(r\right)\right)-\lambda 1_{\left[0,\varepsilon \right]}\left(r\right)w\left(r\right)=-1,\;0\leq r<R,\\ \frac{dw}{dr}\left(R\right)=0, \end{array}$$

where *d* = 2 when the B molecule diffuses within a circular patch of membrane (2.5D and 2D models) and *d* = 3 when the B molecule diffuses within a spherical volume of cytosol (3D model). A no-flux boundary condition is used to prevent the B molecule from leaving the circle (sphere), and we assume that *w*(0) is finite (because the MRT should be finite even if the molecules start at the same location).

*λ* and *ε* are calculated by matching the total volume and the first moment of *σ*_3D_ for the 2.5D and 3D models. That is, given *k*_cat_ and *L*, we choose *λ* and *ε* such that

(5) $$k_{\text{cat}}\int_{0}^{\infty }\sigma_{3\text{D}}\left(r;L\right)r^{n}\;dr=\lambda \int_{0}^{\infty }1_{\left[0,\varepsilon \right]}\left(r\right)r^{n}\;dr,n=2,3.$$

We find that

(6) $$\varepsilon =\alpha L,\;\lambda =\frac{k_{\text{cat}}}{\frac{4}{3}\pi \varepsilon^{3}}=\frac{k_{\text{cat}}}{\frac{4}{3}\pi {\left(\alpha L\right)}^{3}},$$

where $\alpha =16/\left(3\sqrt{6\pi }\right)$.

When using *σ*_2D_ in the 2D model, *λ* and *ε* are calibrated by matching the total area and first moment; see Eqs. S20 and S21. In both calibrations we find that *ε* ∝ *L* (Eqs. 6 and S21), so that in the remainder, we will interchangeably discuss changing *ε* or *L*.

We will focus on the well-mixed MRT, 〈T〉: the average time for the two molecules to react assuming the B molecule is initially placed randomly within the circle (sphere). It is given by

(7) $$\langle T\rangle =\frac{d}{R^{d}}\int_{0}^{R}w\left(r\right)r^{d-1}\;dr.$$

The 2.5D well-mixed MRT, 〈T〉, corresponding to substituting the solution of Eq. 4 into Eq. 7, is given by

(8) $$\langle T\rangle =\{\begin{array}{ll} \frac{1}{\lambda }+\frac{1}{\lambda }[1+\left(\frac{{\hat{R}}^{2}-{\hat{R}}^{2}\rho^{2}}{2\hat{R}\rho }\right)\frac{I_{0}\left(\hat{R}\rho \right)}{I_{1}\left(\hat{R}\rho \right)}]\left(1-\rho^{2}\right)-\frac{R^{2}}{8D}[\rho^{4}-4\rho^{2}+3+4\text{ln}\left(\rho \right)], & \rho \leq 1,\\ \frac{1}{\lambda }, & \rho >1, \end{array}$$

where *ρ* = *ε*/*R* and $\hat{R}=R\sqrt{\lambda /D}$.

To ensure that the replacement of the Gaussian interaction with the indicator function and immobility of the A molecule do not qualitatively change the behavior of the system, we compared Eq. 8 to a 2.5D CRDME model in which both molecules diffuse and react through *σ*_3D_. We demonstrate in Supporting Materials and Methods, Sections S2 and S3 that 〈T〉 obtained from solutions of the Doi model (Eq. 4) gives good qualitative agreement with the results of these CRDME SSA simulations.

To further simplify Eq. 8, we note that *L*/*R* is small in the biologically relevant parameter regime, so *ρ* = *ε*/*R* = *αL*/*R* is also small. For ρ≪1, we therefore expand Eq. 8 in *ρ* to obtain

(9) $$\langle T\rangle ∼\frac{1}{\lambda \rho^{2}}-\frac{R^{2}}{4D}\left(2\text{ln}\left(\rho \right)+1\right)+O\left(\rho^{2}\right),\;\rho \to 0.$$

Using the calibrated parameters in Eq. 6, the 2.5D well-mixed MRT 〈T〉 can then be summarized by

(10) $$\langle T\rangle \{\begin{array}{ll} ∼\frac{\frac{4}{3}\pi R^{2}}{k_{\text{cat}}}\left(\alpha L\right)-\frac{R^{2}}{4D}\left(2\text{ln}\left(\frac{\alpha L}{R}\right)+1\right), & \frac{\alpha L}{R}\ll 1,\\ =\frac{\frac{4}{3}\pi {\left(\alpha L\right)}^{3}}{k_{\text{cat}}}, & \frac{\alpha L}{R}>1. \end{array}$$

Using a similar approach to the preceding analysis (see Supporting Materials and Methods, Sections S4 and S5), the well-mixed MRT of the Doi model (Eq. 4) can be found analytically for both of the 2D (Eq. 8, with calibration given by Eq. S21) and 3D (Eq. S16 with calibration given by Eq. 6) models. Their corresponding asymptotic expansions for ρ≪1 are given by Eqs. S19 and S22.

In summary, we find that over the physical range of molecular reach values, the exact solutions for 〈T〉 from the 2.5D, 2D, and 3D Doi models can be approximated by the asymptotic expansions

(11a, b, c) $$\langle T\rangle ∼\left\{ \begin{array}{ll} \frac{\frac{4}{3}\pi R^{2}}{k_{\text{cat}}}\left(\alpha L\right)-\frac{R^{2}}{4D}\left(2\;\ln\left(\frac{\alpha L}{R}\right)\right)-\frac{R^{2}}{4D}, & \left(2.5\text{D}\right)\\ \frac{\pi R^{2}}{k_{\text{cat}}}-\frac{R^{2}}{4D}\left(2\;\ln\left(\frac{\mu L}{R}\right)\right)-\frac{R^{2}}{4D}, & \left(2\text{D}\right)\\ \frac{\frac{4}{3}\pi R^{3}}{k_{\text{cat}}}+\frac{2R^{3}}{5D}\frac{1}{\alpha L}-\frac{3R^{2}}{5D}, & \left(3\text{D}\right) \end{array}\right.$$

where $\mu =\sqrt{3\pi /8}$. As shown in the Supporting Materials and Methods, for physiological values of *L* and *D*, these expansions agree well with numerical solutions to this model when using the original Gaussian interactions instead of the Doi indicator functions; see Fig. S6.

Fig. 4 plots the three asymptotic expansions as *L* and *D* are varied. Similar to our earlier models, in the physiological 2.5D case (Fig. 4 *B*), we again see that when the diffusivity is small, the reaction is most effective (〈T〉 is smallest) for large values of the molecular reach, whereas for large diffusivities, the reaction is most effective for small values of the molecular reach. In contrast, we observe that in both the 2D (Fig. 4 *E*) and 3D (Fig. 4 *H*) models, increasing the reach always increases the reaction efficacy (decreases 〈T〉). We confirmed the latter result by simulating the biochemical model of the previous section in the fully 3D setting (molecules diffuse in 3D and interact using the 3D kernel), showing that like the Doi model prediction, the potency of the kinase can only increase as the molecular reach increases (Fig. S3).

As we show in the Supporting Materials and Methods, Section S6, the first two terms in each of the three asymptotic expansions have a simple physical interpretation. We can write

(12) $$\langle T\rangle ∼\{\begin{array}{c} \frac{32}{9\pi }\langle T_{\text{RL}}^{\left(2.5\text{D}\right)}\rangle +\langle T_{\text{DL}}^{\left(2.5\text{D}\right)}\rangle -\frac{R^{2}}{4D},\\ \langle T_{\text{RL}}^{\left(2\text{D}\right)}\rangle +\langle T_{\text{DL}}^{\left(2\text{D}\right)}\rangle -\frac{R^{2}}{4D},\\ \langle T_{\text{RL}}^{\left(3\text{D}\right)}\rangle +\frac{6}{5}\langle T_{\text{DL}}^{\left(3\text{D}\right)}\rangle -\frac{3R^{2}}{5D}. \end{array}$$

Here, $\langle T_{\text{RL}}\rangle$ denotes the reaction-limited well-mixed MRT, corresponding to the well-mixed MRT when diffusion is assumed to be infinitely fast; see Eq. S23. $\langle T_{\text{DL}}\rangle$ denotes the leading-order asymptotic expansion of the diffusion-limited well-mixed MRT for ε/R≪1; see Eq. S24. This corresponds to the diffusion-limited regime, in which the molecules are assumed to react instantly upon reaching a separation of *ε*. We therefore see that the well-mixed MRT 〈T〉 can be (approximately) interpreted as the average time for the two molecules to get close enough to react ($\langle T_{\text{DL}}\rangle$) added to the average time for the two molecules to react when diffusion is sufficiently fast that the B molecule is always well-mixed ($\langle T_{\text{RL}}\rangle$).

The regime in which 〈T〉 can increase as *L* increases only arises in the physiological 2.5D model. It is due to the reaction-limited well-mixed MRT, $\langle T_{\text{RL}}^{\left(2.5\text{D}\right)}\rangle$, which is proportional to *L*. Eq. S23 shows that in both the 2D and 3D models, the reaction-limited well-mixed MRT is always independent of *L*, whereas Eq. S24 shows that the leading-order diffusion-limited well-mixed MRTs are decreasing in *L* for any diffusivity in all three models. The scaling of $\langle T_{\text{RL}}^{\left(2.5\text{D}\right)}\rangle$ in *L* results from the use of a 3D Gaussian interaction (with units of inverse volume) in a planar region (with units of area), resulting in an effective well-mixed bimolecular reaction rate *k*_RL_ that scales like *L*^−1^. Because $\langle T_{\text{RL}}^{\left(2.5\text{D}\right)}\rangle =\pi R^{2}/k_{\text{RL}}$, we find that $\langle T_{\text{RL}}^{\left(2.5\text{D}\right)}\rangle \propto L$ (see Supporting Materials and Methods, Section S6 for details).

We can interpret the (physical) differences between the diffusion- and reaction-limited regimes as follows. The diffusing molecule is initially placed randomly but, in the limit of very slow diffusion, is effectively stationary. Let the initial separation between the two reactants be *r*. The probability the reactive sites are in contact is then maximized for *L* = *O*(*r*) in both *σ*_3D_ and *σ*_2D_. If L≪r, the cytoplasmic tails will be too short to contact each other; see Fig. S7 *A*. If L≫r, the tails will explore a large region of space and rarely encounter each other; see Fig. S7 C. When the domain size is much larger than the reach, most initial positions of the slowly diffusing reactant will have r≫L. As such, increasing the reach would be expected to reduce the average of the MRT over the domain (which, by definition, is the well-mixed MRT).

In the limit of very fast diffusion, we think of the diffusing reaction partner as always existing in a uniform probability cloud. The overall reaction process is like a first-order reaction undergone by the stationary reactant, with effective rate constant *k*_eff_. *k*_eff_ is given by the product of two factors. The first is the probability the diffusing reactant is sufficiently close to the stationary reactant to react, i.e., within *ε* = *O*(*L*) of the stationary reactant in the Doi model. Because the diffusing reactant is well-mixed, this probability scales like *L*^2^ when diffusing within the membrane and like *L*^3^ when diffusing in three dimensions. The second factor is the probability per time the molecules can react once sufficiently close, given by *λ* in the Doi model. For *σ*_3D_, the latter scales like *L*^−3^ (see Eq. 6), whereas for *σ*_2D_, the latter scales like *L*^−2^ (see Eq. S21). These scalings reflect the effective region over which the (equilibrated) tails must search for each other once the proteins are sufficiently close, with size *O*(*L*^3^) in the 2.5D and 3D models and size *O*(*L*^2^) in the 2D model. *k*_eff_ is therefore constant in the 2D and 3D models while scaling like *L*^−1^ in the 2.5D model (and hence, the well-mixed MRT will scale like $k_{\text{eff}}^{-1}=O\left(L\right)$, as observed in Fig. 4 *C*). We therefore see that in the reaction-limited regime, we can interpret the behavior of the reaction time as being a balance between an exploration effect of the two proteins (the two molecules are close enough to react, an increasing function of *L*) and a dilution effect (the effective concentration of the reactive site complex within the region explored by the tails, a decreasing function of *L*).

In summary, we find that for tethered signaling reactions, the reaction time (i.e., 〈T〉) can exhibit a different functional dependence on molecular reach over physiological parameter regimes when diffusion is fast versus slow. This arises from having 3D interactions between cytoplasmic tails of molecules confined to diffuse within a 2D membrane (2.5D model). We also find that when diffusion is sufficiently fast, the reaction time is independent of *L* for molecules diffusing and reacting in 3D (3D model) or diffusing and reacting purely in 2D (2D model). In contrast, the reaction time is still dependent on *L* for molecules diffusing in the membrane but reacting through the 3D interaction kernel (2.5D model). This illustrates how molecular reach in tethered signaling can reduce potency in 2D but not 3D geometries.

## Discussion

Using a combination of spatial simulations and analytical calculations, we have examined the influence of molecular reach on membrane-confined reactions. Our key finding is that increases in molecular reach can increase reaction rates (or receptor potency) when diffusion is slow but decrease reaction rates (or receptor potency) when diffusion is fast. This switch is critically dependent on molecules diffusing in 2D but explicitly allowing them to react in the 3D volume proximal to the membrane using a 3D reaction kernel. The work underlines the importance of the 3D nature of 2D membrane-confined reactions.

### Reactions in 2D versus 3D

It is an open problem to understand how membrane confinement modulates receptor-ligand binding and biochemical reactions. Mathematical models of membrane reactions commonly restrict molecules to not only diffuse in 2D but to react through 2D interactions (23, 26, 27, 28, 29). Although transmembrane domains (e.g., that localize PD-1 and CD28) and membrane-anchoring modifications (e.g., palmitoylation that localizes LCK) restrict molecules to diffuse in the 2D membrane, their tethers allow them to explore a 3D cytoplasmic volume that is proximal to it. The switch in efficacy that we report critically relied on explicitly accounting for this through a physiological 3D kernel; using an idealized 2D kernel that forced molecules to interact within the plane of the membrane did not produce the switch.

### Modeling 3D reaction kernels for 2D membrane reactions

We have explored the molecular reach of the reaction primarily using a stationary Gaussian reaction kernel inspired by the WLC polymer model. It is likely that in some biological situations, the polymer does not equilibrate quickly (stationary assumption) and/or the kernel is not Gaussian. We calculated that the stationary assumption is valid in our simulations (see Supporting Materials and Methods, Section S9), but this assumption will break down if, for example, longer tethers are simulated. A Gaussian kernel is expected to accurately capture the molecular reach of freely diffusing unstructured polypeptide chains such as the unstructured cytoplasmic tails of immune receptors (5). However, there is evidence that the cytoplasmic tails of NTRs, including CD28, may have regulated interactions with the plasma membrane (30, 31, 32, 33), which may lead to a non-Gaussian kernel. Similarly, a Gaussian kernel is expected to only be an approximation when applied to structured proteins like SHP-1/SHP-2 that contain multiple domains connected by flexible linkers. We note that experimental data of tethered dephosphorylation by SHP-1 were well-fitted by a Gaussian kernel (10). Nonetheless, careful consideration is needed when formulating a 3D reaction kernel, and it may be feasible to determine the kernel using molecular dynamics or coarse-grained mesoscale simulations (34) that can be adapted to the specific molecules of interest.

It should also be noted that we have not considered biological contexts in which all reactants involved in a tethered signaling reaction are present at high densities. For sufficiently large concentrations, our general observations concerning the influence of molecular reach on reaction statistics could potentially change. Such density-dependent results were recently observed in a model for transport through the nuclear pore, in which a continuum of elastic tethers was shown to potentially hinder diffusive particle motion for small numbers of molecules while enhancing particle motion at sufficiently large densities (35). At high densities, steric effects have also been shown to influence clustering of membrane proteins interacting through tethered reaction processes (36). We note that our first model, of PD-1 inhibition of CD28, used physiological estimates for CD28 concentration while varying PD-1 concentration (37).

### Implications for the biology of immune receptors

The ability of receptors within the NTR or immunoreceptor group (5) to regulate the phosphorylation of specific substrates is dependent on the signaling protein recruited by the receptor (e.g., SHP-2 in the case of PD-1), the specificity of the signaling protein to the specific substrate (e.g., SHP-2 has the ability to dephosphorylate CD28 (7)), and the ability of the receptor and substrate to localize (e.g., PD-1/CD28 coclustering (21)). In addition to these mechanisms, our work demonstrates that the molecular reach of a reaction may also control the ability of a receptor to regulate the phosphorylation state of the substrate and hence determine receptor potency. A key question that this work raises is whether increasing the molecular reach will increase or decrease receptor potency. Although PD-1 and CD28 are expected to be mobile on resting T cells, their relative mobility within ligand-induced clusters has yet to be investigated. Our work indicates that increasing the molecular reach of this reaction will only increase PD-1 potency if their mobility is reduced within these ∼100 nm clusters (21).

### Experimental measurements

Tethered signaling depends on binding, catalysis, and the molecular reach of the reaction. Although standard assays are available to study binding (e.g., surface plasmon resonance (38)) and catalysis (e.g., reaction product measurements in solution (39)), it is more challenging to produce a physiologically relevant assay to explore the role of molecular reach. Recently, an in vitro reconstitution of the dephosphorylation of CD28 by PD-1 has been described whereby CD28 and PD-1 were localized to the two-dimensional surface of liposomes (7). This system can be used to experimentally determine how changes to the molecular reach of the reaction influence the potency of PD-1. We have also recently introduced a surface plasmon resonance-based assay that can directly determine the molecular reach for fratricide reactions (10). As these experimental tools mature, it may become feasible to systematically examine the role of molecular reach in controlling tethered signaling reactions.

### Molecular reach beyond potency

In this work, we have focused on the role of molecular reach in modulating reaction efficacy or potency. Given that phosphorylation reactions, and noncovalent post-translational modifications more generally, have been shown to give rise to a variety of information processing phenotypes, it would be interesting to examine the impact of molecular reach in these contexts (25, 40). For example, phosphorylation reactions are known to produce ultrasensitive or switch-like responses by multisite phosphorylation (23, 41, 42, 43, 44, 45), but processivity, whereby an enzyme modifies multiple sites per collision, can reduce or even abolish ultrasensitivity (23, 46). Given that molecular reach can allow enzymes to catalyze reactions at a distance, it may effectively generate processive enzymes that can modulate ultrasensitivity. It would be interesting to examine how molecular reach controls other features of signaling in the future.

## Supporting Citations

References (47, 48, 49, 50, 51, 52, 53, 54, 55, 56, 57, 58, 59, 60, 61, 62, 63, 64) appear in the Supporting Material.

## Supporting Material

Document S1. Supporting Materials and Methods, Figs. S1–S10, and Table S1

Document S2. Article plus Supporting Material
