# Supplementary material for: The Influence of Molecular Reach and Diffusivity on the Efficacy of Membrane-Confined Reactions
Source: Biophys J. 2019 Aug 28;117(7):1189–201. doi: 10.1016/j.bpj.2019.08.023 (PMC6818170; doi:10.1016/j.bpj.2019.08.023)
Supplement: Document S1. Supporting Materials and Methods, Figs. S1–S10, and Table S1 [file mmc1.pdf]

Biophysical Journal, Volume *117*

Supplemental Information

The Influence of Molecular Reach and Diffusivity on the Efficacy of Membrane-Confined Reactions

Ying Zhang, Lara Clemens, Jesse Goyette, Jun Allard, Omer Dushek, and Samuel A. Isaacson

Supporting Material: The influence of molecular reach and diffusivity on the efficacy of membrane-confined reactions

Ying Zhang[1], Lara Clemens[2], Jesse Goyette[3], Jun Allard[2], Omer Dushek[4,*], and Samuel. A. Isaacson[1,*]

[1]Boston University, Department of Mathematics and Statistics, Boston, MA 02215
[2]Center for Complex Biological Systems, University of California - Irvine, Irvine, CA
[3]School of Medical Sciences, University of New South Wales, Sydney 2052, Australia
[4]Sir William Dunn School of Pathology, University of Oxford, Oxford, OX1 3RE, U.K.

S1 Introduction to CRDME Model and Simulation Algorithm

The convergent reaction-diffusion master equation (CRDME) model we use corresponds to a spatial discretization of the general volume-reactivity model. In the latter, molecules are represented as point particles moving by Brownian motion[(1–3)]. First order reactions are modeled as internal processes with Poisson clocks, while bimolecular reactions between two molecules occur with a separation dependent probability per time (given by the Gaussian function $k_{\text{cat}}\sigma(r; L)$ for separation r, catalytic rate k_{cat}, and molecular reach L, see Methods). These mathematical models can be described by their corresponding forward Kolmogorov equation, a high-dimensional coupled system of partial-integral differential equations for the probability density of having a given number of each chemical species at specified locations at a given time[(2,3)]. For example, Eq. S13 is the forward Kolmogorov equation for the simplified case of just two molecules that can annihilate through the Gaussian interaction.

For multiparticle systems, the high-dimensionality of these equations precludes their solution by standard numerical methods for solving PDEs. Instead, we approximate the stochastic process of the individual molecules diffusing and reacting. In this work we do so by first spatially discretizing the forward Kolmogorov equation of the volume-reactivity model to a continuous-time Master Equation defined on a Cartesian mesh. We call this spatially discrete model the convergent reaction-diffusion master equation (CRDME)[(3,4)]. As the set of ODEs that comprise the CRDME are still too high-dimensional to solve numerically, we instead generate exact realizations of the corresponding jump process associated with the CRDME using the Gibson-Bruck SSA method[(5)] (a variant of the well-known Gillespie method[(6,7)]). We will subsequently call this simulation method the CRDME SSA. Here the diffusion of individual molecules is approximated by a continuous time random walk of the molecules hopping between voxels of the Cartesian mesh. Bimolecular reactions between reactant molecules in nearby voxels occur with probabilities per time derived from $\sigma(\cdot; L)$, see[(3,4)] for full details.

As an illustrative example, consider a system with three chemical species, $\{\mathrm{A}, \mathrm{B}, \mathrm{C}\}$, with each molecule diffusing within a square with periodic boundary conditions. Assume all molecules have diffusivity D, and the molecules

*Correspondence: isaacson@math.bu.edu or omer.dushek@path.ox.ac.uk.

Table S1: Example of diffusive and chemical transitions in CRDME SSA simulations

	Transitions	Transition Rates (units of per time)	Upon Transition Event
Diffusive hopping from V_j to neighbor $V_i \in \mathcal{N}(V_j)$:	$\text{A}_j \to \text{A}_i$	$DA_j(t)/h^2$	$A_i := A_i + 1, A_j := A_j - 1,$
	$\text{B}_j \to \text{B}_i$	$DB_j(t)/h^2$	$B_i := B_i + 1, B_j := B_j - 1,$
	$\text{C}_j \to \text{C}_i$	$DC_j(t)/h^2$	$C_i := C_i + 1, C_j := C_j - 1,$
Chemical Reactions:	$\text{A}_i + \text{B}_j \to \text{C}_i + \text{B}_j$	$\alpha_{ij} A_i(t) B_j(t)$	$A_i := A_i - 1, C_i := C_i + 1.$
	$\text{C}_i \to \text{A}_i$	$\lambda C_i(t)$	$A_i := A_i + 1, C_i := C_i - 1.$

For the $\text{C} \xrightarrow{\lambda} \text{A}$ and $\text{A} + \text{B} \xrightarrow{k_{\text{cat}}\sigma(r;L)} \text{C} + \text{B}$ reaction-diffusion system, the table shows the five basic types of jump process transitions that can occur. Here V_j labels a given voxel of the Cartesian mesh, with mesh width h and four nearest-neighbors $\mathcal{N}(V_j)$. $A_j(t)$, $B_j(t)$ and $C_j(t)$ denote the stochastic processes for the number of molecules of each species in the jth voxel at time t. See SI S1 for details.

may undergo the reactions $\text{C} \xrightarrow{\lambda} \text{A}$ and $\text{A} + \text{B} \xrightarrow{k_{\text{cat}}\sigma(r;L)} \text{C} + \text{B}$ (where r denotes the separation of an individual pair of A and B molecules). To derive the CRDME model, we discretize the square into a collection of N square mesh voxels, $\{V_i\}_{i=1}^{N}$, of width h. Let $\mathcal{N}(V_i)$ label the set of the four nearest-neighbor voxels to voxel V_i. The CRDME SSA then simulates the set of possible jump process transitions shown in Table S1. The bimolecular reaction transition rate in the table (i.e. probability per time) for one specific A molecule in V_i and one specific B molecule in V_j to react is given by

$$\alpha_{ij} = \frac{k_{\text{cat}}}{h^4} \int_{V_i} \int_{V_j} \sigma(|\boldsymbol{x} - \boldsymbol{y}|\,; L)\, d\boldsymbol{x}\, d\boldsymbol{y},$$

as derived in[3].

S2 Derivation of the Doi model

We show here how to derive the simplified Doi model used in the Results section, beginning with a simplified model in which both the A and B molecules diffuse and interact through a Gaussian kernel. We again consider the two-particle annihilation reaction

$$\text{A} + \text{B} \xrightarrow{k_{\text{cat}}\sigma(r;L)} \emptyset.$$

Assume the two molecules diffuse within a square (2.5D or 2D) or a cube (3D), Ω, where the length of each edge of the square (cube) is 300nm. Denote by $p(\boldsymbol{x}, \boldsymbol{y}, t)$ the probability density an A molecule at $\boldsymbol{x} \in \Omega$ and a B at $\boldsymbol{y} \in \Omega$ have not yet reacted at time t. We consider the volume-reactivity model,

$$\begin{aligned} \frac{\partial p}{\partial t} &= D(\Delta_x + \Delta_y) p(\boldsymbol{x}, \boldsymbol{y}, t) - k_{\text{cat}} \sigma(|\boldsymbol{x} - \boldsymbol{y}|\,; L) p(\boldsymbol{x}, \boldsymbol{y}, t), \\ p(\boldsymbol{x}, \boldsymbol{y}, 0) &= \frac{1}{|\Omega|^2}, \end{aligned} \tag{S13}$$

with reflecting boundary conditions on the boundary, $\partial\Omega$, in each of $\boldsymbol{x}$ and $\boldsymbol{y}$. Here k_{cat} is the catalytic rate of the reaction and L the molecular reach. $|\Omega|$ denotes the area (or volume) of Ω, so that the initial condition corresponds to starting both molecules well-mixed (i.e. uniformly distributed) within Ω.

We will focus on the behavior of the well-mixed mean reaction time (MRT), i.e. the average time for the diffusing A and B molecules to react assuming they are each placed randomly within Ω. Let T denote the random time at which the A and B molecules react. The exact well-mixed MRT is then given by the average of T, which we denote by $\langle T \rangle$. It is defined by

$$\langle T \rangle = \int_0^\infty \int_\Omega \int_\Omega p(\boldsymbol{x}, \boldsymbol{y}, t)\, d\boldsymbol{x}\, d\boldsymbol{y}\, dt. \tag{S14}$$

We simulate an approximation to the stochastic process of the two molecules diffusing and reacting using the CRDME SSA (see Methods and SI Section S1). Fig. S4 shows the estimated well-mixed MRT from 50000 CRDME SSA simulations in 2.5D. Observe that this figure is qualitatively quite similar to the asymptotic approximation to $\langle T \rangle$ found for the Doi model, Fig. 4B.

We now introduce two simplifications to the preceding model to obtain the Doi model. Due to presence of the 3D Gaussian interaction term $\sigma(\cdot; L)$ in Eq. S13, analytically solving for the exact $\langle T \rangle$ satisfying Eq. S14 is impractical. We therefore simplify Eq. S13 by transforming to a radially symmetric problem on a circle (sphere) of equivalent area (volume). In this reduced model one molecule is assumed stationary at the origin, while the other molecule diffuses with relative diffusivity D within a circle (sphere) of radius R about the origin. The MRT $u(r)$ for a diffusing molecule that is initially placed a distance r from the origin satisfies

$$\begin{aligned} \frac{D}{r^{d-1}} \frac{d}{dr}\left(r^{d-1}\frac{du}{dr}(r)\right) - k_{\text{cat}}\sigma(r;L)u(r) &= -1, \quad 0 \le r < R, \\ \frac{du}{dr}(R) &= 0, \end{aligned} \tag{S15}$$

where $d = 2$ for the 2.5D and 2D models, and $d = 3$ for the 3D model. In each case we also assume $u(0)$ is finite, since the time for the two-molecules to react should remain finite even when their initial positions are the same. The corresponding well-mixed MRT when the position of the diffusing molecule is initially uniformly distributed is then given by

$$\langle T \rangle = \frac{d}{R^d} \int_0^R u(r) r^{d-1}\, dr,$$

where again, $d = 2$ for the 2.5D and 2D models, and $d = 3$ for the 3D model. Eq. S15 is easily solved by a standard finite volume discretization, described in SI Section S3. Fig. S5 (solid lines) shows that in 2.5D this model gives almost identical results to the CRDME SSA approximation of Eq. S13 shown in Fig. S4.

To obtain an explicit analytical approximation to the solution of Eq. S15, we perform one final transform. We approximate Eq. S15 by a Doi model. As described in the Results section, the Doi model replaces the Gaussian interaction $\sigma(r; L)$ by an approximating indicator function $\lambda \mathbb{1}_{[0,\varepsilon]}(r)$, giving the final model in Eq. 4. In the Results Section and SI Section S5 we describe how λ and ε are calibrated for all three cases. In the next section we show that the exact solutions in 2.5D, 2D and 3D agree well with the corresponding numerical solutions to the Gaussian interaction model (Eq. S15) over physiological parameter ranges.

S3 Solving the radially (spherically) symmetric problem on a circle (sphere)

A key step in the analysis of the simplified model is to approximate the two-particle problem (Eq. S13) on a square (cube) with reflecting boundary conditions by a radially (spherically) symmetric problem (Eq. S15) with the same area (volume) and a reflecting boundary condition. Fig. S5A shows that the numerical solution of the PDE in Eq. S15 (solid lines) preserves the behavior of the 2.5D well-mixed MRT for the annihilation reaction obtained by CRDME SSA simulations of Eq. S13 (Fig. S4). We solved Eq. S15 numerically using a standard second-order finite volume discretization (in both the circle and sphere).

Similarly, in Eq. 4 we replace the Gaussian interaction of Eq. S15 by a calibrated Doi-model step-function interaction (see Eqs. 6 and S21). Fig. S5 shows that the exact solutions to the calibrated Doi model in Eq. 4 (dashed lines) in 2.5D (Fig. S5A), 2D (Fig. S5B), and 3D (Fig. S5C), each agree well with the numerical solution of the Gaussian interaction model (solid lines).

S4 3D well-mixed MRT for simplified Doi model

Using the same approach as for the 2.5D MRT analysis (see Results and S2), we map Eq. S15 to a 3D Doi type model with calibrated parameters that is analytically solvable. The 3D well-mixed MRT, $\langle T \rangle$, corresponding to substituting the solution of Eq. 4 into Eq. 7, is given by

$$\langle T \rangle = \begin{cases} F(\rho), & \rho \leq 1, \\ \frac{1}{\lambda}, & \rho \geq 1, \end{cases} \tag{S16}$$

with $\rho = \varepsilon/R$, $\hat{R} = R\sqrt{\lambda/D}$ and

$$F(\rho) \;=\; \frac{1}{\lambda} + \frac{R^2}{15D}\left(\frac{5}{\rho} - 9 + 5\rho^2 - \rho^5\right) + (1 - \rho^3)\left[\frac{1}{\lambda} + \frac{(R^3 - R^3\rho^3)}{3DR\rho}\left(\frac{\tanh(\hat{R}\rho)}{\hat{R}\rho - \tanh(\hat{R}\rho)}\right)\right]. \tag{S17}$$

We use the same parameter calibration (Eq. 5) as we used for the 2.5D Doi model (Eq. 4) with 3D Gaussian interaction. ε and λ are then given by Eq. 6. Expanding Eq. S16 in ρ for $\rho \ll 1$ we find

$$\langle T \rangle \sim \frac{1}{\lambda}\frac{1}{\rho^3} + \frac{2R^2}{5D}\frac{1}{\rho} - \frac{3R^2}{5D} + \mathcal{O}(\rho). \tag{S18}$$

Substituting in the calibrated values for λ and ε then gives

$$\langle T \rangle \begin{cases} \sim \frac{\frac{4}{3}\pi R^3}{k_{\text{cat}}} + \frac{2R^3}{5D}\frac{1}{\alpha L} - \frac{3R^2}{5D}, & \frac{\alpha L}{R} \ll 1, \\ \\ = \frac{\frac{4}{3}\pi(\alpha L)^3}{k_{\text{cat}}}, & \frac{\alpha L}{R} > 1, \end{cases} \tag{S19}$$

where $\alpha = 16/(3\sqrt{6\pi})$.

S5 2D well-mixed MRT for a 2D concentration kernel

A key feature in all our models of tethered signalling is the use of a 3D Gaussian concentration kernel $\sigma(r; L)$ to determine interaction functions for bimolecular reactions in the membrane. We now consider how the well-mixed MRT in the simplified 2.5D model (Eq. S15) changes if we instead use the 2D Gaussian interaction kernel $\sigma_{\text{2D}}(r; L)$ defined in Eq. 2. We call this new model the 2D model. We follow a similar analysis as in the preceding section and the Results.

The 2D well-mixed MRT, $\langle T \rangle$, obtained by substituting the solution of Eq. 4 into Eq. 7 is given by Eq. 8. As we now consider a 2D Gaussian interaction, we match the total area (equivalently total reaction rate) and the first moment of the 2D Gaussian using

$$k_{\text{cat}} \int_0^\infty \sigma_{\text{2D}}(r; L) r^n \, dr = \lambda \int_0^\infty \mathbb{1}_{[0,\varepsilon]}(r) r^n \, dr, \quad n = 1, 2, \tag{S20}$$

to obtain

$$\varepsilon = \mu L, \qquad\qquad \lambda = \frac{k_{\text{cat}}}{\pi\varepsilon^2} = \frac{k_{\text{cat}}}{\pi(\mu L)^2}, \tag{S21}$$

where $\mu = \sqrt{3\pi/8}$. Notice, we now see the key difference from the use of a 3D interaction kernel; λ now scales like L^{-2} instead of L^{-3} as we previously found.

Expanding Eq. 8 in ρ for $\rho \ll 1$ we again have Eq. 9, which combined with the preceding calibration for ε and λ then gives

$$\langle T \rangle \begin{cases} \sim \frac{\pi R^2}{k_{\text{cat}}} - \frac{R^2}{4D}\left(2\ln\left(\frac{\mu L}{R}\right)+1\right), & \frac{\mu L}{R} \ll 1, \\ = \frac{\pi(\mu L)^2}{k_{\text{cat}}}, & \frac{\mu L}{R} > 1. \end{cases} \tag{S22}$$

S6 The well-mixed mean reaction time from the Doi model is approximately the sum of the reaction– and diffusion–limited mean reaction times

In each of the three asymptotic expansions Eqs. 11a, 11b and 11c, we now show the first two terms have a simple physical interpretation. The first term is essentially the mean reaction time if the system were reaction-limited (i.e. the well-mixed mean reaction time when $D = \infty$). The second term is essentially the leading order diffusion-limited mean reaction time (i.e. the well-mixed mean reaction time when the reaction occurs instantly once the reactants are sufficiently close). We will illustrate how the former is responsible for the scaling regime where $\langle T \rangle$ grows in L for the (physiological) 2.5D model.

We first consider the well-mixed mean reaction time in the reaction limited regime. Assume that the diffusivity D of the diffusing molecule is infinite, so that the system is completely well-mixed. The position of the diffusing molecule is then given by a uniform density,

$$\bar{p}(r) = \bar{p} = \begin{cases} \frac{1}{\pi R^2}, & \text{in 2D}, \\ \frac{1}{\frac{4}{3}\pi R^3}, & \text{in 3D}. \end{cases}$$

For each of the 2.5D, 2D and 3D models considered in the last Results section, in this regime we expect the total probability per time the molecules react to be given in terms of a well-mixed reaction-limited reaction rate, k_{RL}, by

$$k_{\text{RL}}\bar{p} = \begin{cases} k_{\text{cat}}(2\pi)\int_0^R \sigma_{\text{3D}}(r;L)\bar{p}\, r\, dr, & \text{2.5D} \\ k_{\text{cat}}(2\pi)\int_0^R \sigma_{\text{2D}}(r;L)\bar{p}\, r\, dr, & \text{2D} \\ k_{\text{cat}}(4\pi)\int_0^R \sigma_{\text{3D}}(r;L)\bar{p}\, r^2\, dr, & \text{3D}. \end{cases}$$

Assuming R is large, the integrals are well-approximated by the integral over $r \in [0, \infty)$, so that

$$k_{\text{RL}}\bar{p} \approx \begin{cases} \sqrt{\frac{3}{2\pi}}\frac{k_{\text{cat}}}{\pi R^2}\frac{1}{L}, & \text{2.5D} \\ \frac{k_{\text{cat}}}{\pi R^2}, & \text{2D} \\ \frac{k_{\text{cat}}}{\frac{4}{3}\pi R^3}, & \text{3D}. \end{cases}$$

We note that the latter two are simply the standard probability per time a well-mixed reaction with bimolecular rate constant k_{cat} occurs within a circle (sphere) of radius R. The inverse of $k_{\text{RL}}\bar{p}$ then defines the reaction-limited well-mixed mean reaction time,

$$\begin{aligned} \langle T_{\text{RL}}^{(2.5\text{D})} \rangle &= \sqrt{\frac{2\pi}{3}}\frac{\pi R^2 L}{k_{\text{cat}}}, \\ \langle T_{\text{RL}}^{(2\text{D})} \rangle &= \frac{\pi R^2}{k_{\text{cat}}}, \\ \langle T_{\text{RL}}^{(3\text{D})} \rangle &= \frac{\frac{4}{3}\pi R^3}{k_{\text{cat}}}. \end{aligned} \tag{S23}$$

Only $\langle T_{\text{RL}}^{(2.5\text{D})} \rangle$ varies with L, increasing linearly as L increases.

Similarly, we may consider a diffusion limited regime in the (calibrated) Doi model, where the molecules react instantly upon the diffusing molecule reaching $r = \varepsilon$. The leading order asymptotic expansions for $\varepsilon/R \ll 1$ of the diffusion limited well-mixed mean reaction time are well-known, see[8,9], and given by

$$\begin{aligned} \langle T_{\text{DL}}^{(2.5\text{D})} \rangle &\sim -\frac{R^2}{2D} \log\left(\frac{\alpha L}{R}\right), \\ \langle T_{\text{DL}}^{(2\text{D})} \rangle &\sim -\frac{R^2}{2D} \log\left(\frac{\mu L}{R}\right), \\ \langle T_{\text{DL}}^{(3\text{D})} \rangle &\sim \frac{R^3}{3D\alpha L}. \end{aligned} \tag{S24}$$

All three diffusion limited mean reaction-times are decreasing as L increases.

We therefore see that the first two terms in the asymptotic expansions of $\langle T \rangle$ from the Doi model (Eqs. 11a, 11b and 11c) can be summarized as essentially a sum of the reaction-limited and diffusion-limited well-mixed mean reaction times as given in Eq. 12.

S7 Domain size dependence of the CD28 model

As a simple control, we solve the CD28 model (see Results) using a larger domain size to confirm that the steady-state fraction of phosphorylated CD28 exhibits a similar qualitative behavior with respect to the diffusivity and molecular reach. We see from Fig. S8C and S8D that solving the model on a larger square of side length of 500nm preserves the qualitative dependence of the steady-state fraction of phosphorylated CD28 on D and L.

S8 Determining termination time in CRDME simulations

To determine the termination time in the CRDME SSA simulations of the first two models of the Results section, for each pair of diffusivity and molecular reach values we ran 100 test simulations. We then estimated an approximate time at which mean concentrations and/or fractions of each chemical species had reached steady state. In our final larger sampling runs (with $O(10^4)$ to $O(10^5)$ simulations per parameter set), we then set the termination time to be 40%-50% larger than the estimated time steady-state was reached. In Fig. S9 we plot till the termination time used in our final simulations the average fraction of phosphorylated CD28 in the CD28 model from 100 simulations (for different values of the diffusivity and molecular reach). In all nine cases the CD28 model appears to have reached steady state well before the chosen termination time.

S9 Validity of using a stationary probability density kernel (σ)

We have used a stationary kernel (σ) to represent the probability that two tethers interact. This assumption is only valid when the timescale over which the polymer tether explores its space (τ) is smaller than the timescale for reaction and diffusion. An approximate upper bound on τ can be obtained by assuming the polymer is a freely-jointed chain with N Kuhn segments of length b, and monomer size $< b$. These are related to the reach parameter, L, by $L = b\sqrt{N/2}$. A lower bound on the diffusion coefficient of the polymer is $6\pi\eta L/k_B T$, leading to the following upper bound estimate,

$$\tau \sim \frac{6\pi\eta}{k_B T} N^{3/2} b^3 = \frac{3\pi\eta}{\sqrt{2} k_B T} L^3, \tag{S25}$$

where η is the viscosity of the surrounding medium. Assuming that the viscosity of the cytoplasm is close to that of water, we find that $\tau \lesssim 10^{-4}$ s for the maximum molecular reach of $L = 32$ nm in Fig. 2. This is smaller than the

fastest diffusive timescale ($\sim L^2/D$ = (32 nm)^2/0.125 μm^2/s = 8×10^{-3} s) and reaction timescale ($\sim k^*_{\text{cat}}(1/L^3)$ = 0.1 μM^{-1}s^{-1} / (32 nm)^3 = 0.2 s).

The above approximation has been derived in a more general setting(10,11) and confirmed in numerical simulation(11–13). This model was quantitatively confirmed and in particular the diffusion coefficient equation(14) and the loop closure time equation(15) were found to be in close agreement. The scaling law $\tau \sim N^{3/2}$ was confirmed experimentally(16) and by all-atom dynamics(17).

S10 Reaction kernel for surface-tethered molecules

As discussed in the Materials & Methods, if two tethers have their bases fixed at a separation distance r, then the probability density for the reaction sites of the tethers to interact is given by Eq. 1, which is referred to as the reaction kernel. This equation was derived by assuming each tether i explores three-dimensional space with Gaussian probability density. We note that this formulation assumed that the tether was allowed to explore all of space. In this section, we ask, what is the interaction kernel $\sigma_{\text{surf}}(r)$ for two tethers with base fixed at a distance r, but which are attached to a 2D surface (i.e. the plasma membrane), and therefore can only explore half-space.

Since surface-adhered polymers do not obey Gaussian probability densities that only depend on L, we must specify more detailed polymer properties. We assume the tethers are freely-jointed chains composed of N rigid segments of length δ. In free-space, this chain has a reach parameter of $L = \sqrt{L_p L_C} = \sqrt{N}\delta/2$. We assume $\delta = 0.3$ nm consistent with previous models of disordered proteins and PEG(18,19). We explore a range of tether lengths $N = 25 - 1000$ corresponding to free-space reach parameters $L = 1.5 - 13$ nm. We simulate the ensemble of polymer conformations of the two freely-jointed chains, for various base separation distances r, using a standard Metropolis method(13,20) and determine the probability that the reaction sites interact to determine σ_{surf}.

In Fig. S10A we show the probability density of the end-to-end distances for various values of L. In all cases, the distances are slightly elongated by the presence of the surface. This is in agreement with previous findings that adherence to a surface tends to elongate polymers(21). In Fig. S10B, we show the reaction kernel $\sigma_{\text{surf}}(r)$. In all cases, we find that the effective concentrations are enhanced by the presence of the surface. This makes intuitive sense, since reaction sites are forced by the surface to be in the same half-space. The length-scale of the separation distance, i.e., the decay length of the curves in Fig. S10B, is approximately unchanged by the surface. The simulation therefore suggests that the reaction kernel for surface-bound tethers is well-approximated by

$$\sigma_{\text{surf}}(r) \approx c_{\text{surf}}\ \sigma_{\text{3D}}(r), \tag{S26}$$

$$= c_{\text{surf}} \left(\frac{3}{2\pi L^2}\right)^{3/2} \exp\left(-\frac{3r^2}{2L^2}\right), \tag{S27}$$

where $c_{\text{surf}} > 1$ is an enhancement factor that arises as the surface forces the tethers together.

By fitting Eq. S27 to the simulated kernels in Fig. S10B, we produce estimates for the enhancement factor c_{surf} in Fig. S10C. We find that $c_{\text{surf}} \approx 1.5$ over the estimated physiological range of L (for 7 nm$< L <$ 13 nm, always within 10%). In CRDME simulations of reactions between tethered molecules, the per-second reaction rate is $k_{\text{cat}}\sigma_{\text{3D}}(r)$. The results here allow us to use the same scheme to simulate reactions between surface-tethered molecules, but with the prefactor k_{cat} reduced by ≈ 1.5.

To further verify the validity of this approximation, in Fig. S10D we plot the re-scaled kernel

$$\frac{\sigma_{\text{3D}}(r)}{\left(\frac{3}{2\pi L^2}\right)^{3/2}} \qquad \text{versus} \qquad \frac{r}{\sqrt{\frac{3}{2L^2}}}. \tag{S28}$$

In this re-scaling, all free-space kernels collapse onto a single curve (black curve in Fig. S10D). We now plot the

surface-adhered kernels re-scaled as

$$\frac{\sigma_{\rm 3D}(r)}{c_{\rm surf}\left(\frac{3}{2\pi L^2}\right)^{3/2}} \qquad \text{versus} \qquad \frac{r}{\sqrt{\frac{3}{2L^2}}}. \tag{S29}$$

We find that these surface-adhered kernels also collapse onto the same single curve. This confirms the approximation given by Eq. S27.

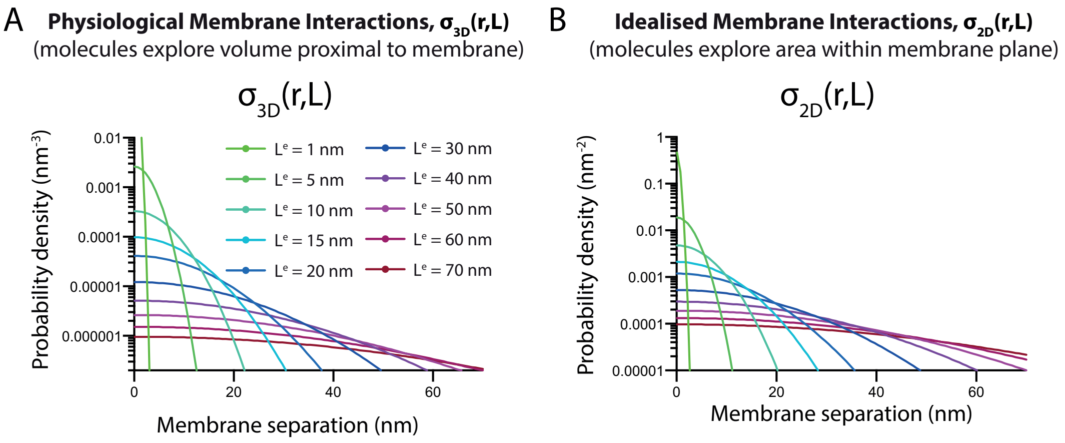

Figure S1: Comparison of A) σ_{3D} and B) σ_{2D} over the membrane separation distance (nm) for the indicated value of the molecular reach of the reaction (L).

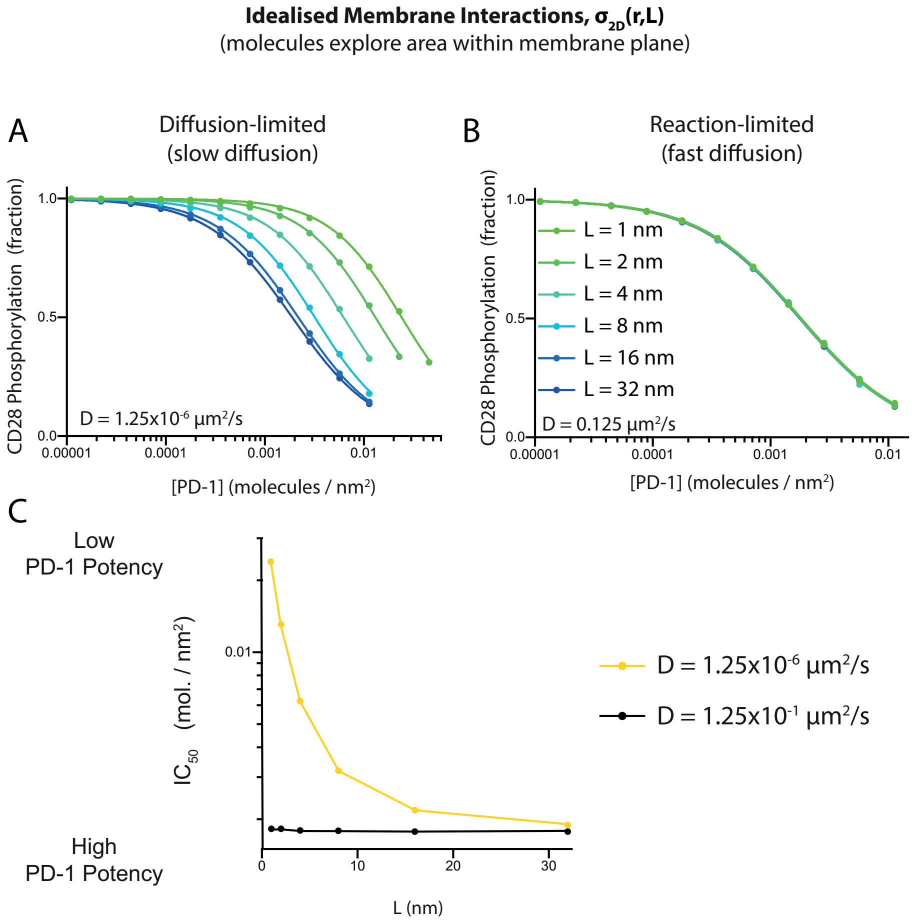

Figure S2: Reproducing Fig. 2 using the idealised 2D interaction kernel σ_{2D} shows that increasing the molecular reach A) increases PD-1 potency in the diffusion-limited regime but B) has no effect in the reaction-limited regime. C) The potency over the molecular reach quantified from A and B.

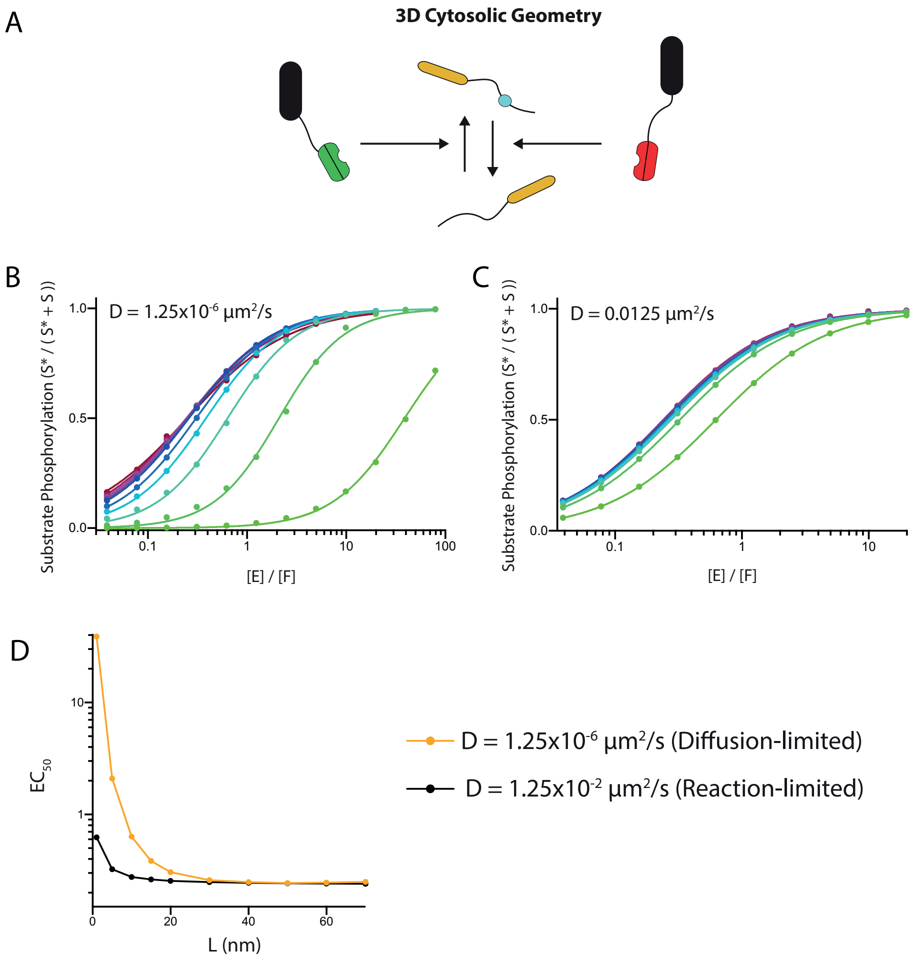

Figure S3: Reproducing Fig. 3A,C,D,E when molecules freely diffuse in 3D using the physiological 3D interaction kernel σ_{3D}. A) Schematic of model highlighting that molecules are not confined to a membrane. B-C) Fraction of phosphorylated substrate in the steady-state for the indicated values of the molecular reach of the reaction when reactions B) are limited by diffusion or C) are not limited by diffusion. D) The ratio of kinase-to-phosphatase that produces half-maximal phosphorylation over the molecular reach of the reaction showing that increasing the molecular reach can only increase potency in this geometry. Parameter values: [S] = $8.5 \times 10^4 \mu\mathrm{m}^{-3}$, [F] = $9.4 \times 10^4 \mu\mathrm{m}^{-3}$, domain size = 300nm $\times$ 300nm $\times$ 300nm, and all other parameters as indicated in Table 2.

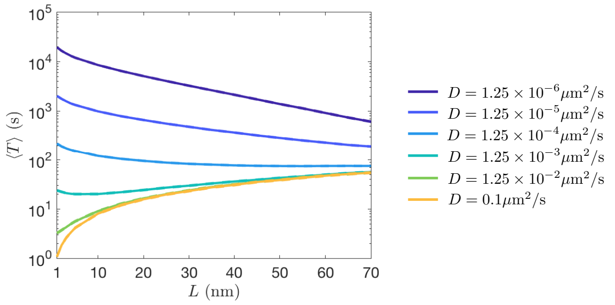

Figure S4: The well-mixed mean reaction time, $\langle T \rangle$, of the two-particle $\mathrm{A} + \mathrm{B} \xrightarrow{k_{\mathrm{cat}}\sigma(r;L)} \emptyset$ reaction changes its dependence on molecular reach for small vs. large diffusivities when the molecules diffuse in a (2D) membrane, but their tails can react in the (3D) cytosol. For each value of D and L we estimated $\langle T \rangle$ from 50000 CRDME-SSA simulations. 95% confidence intervals for each curve are given by dashed lines of the same color (barely visible). The catalytic rate k_{cat} was 0.1 $\mu\mathrm{M}^{-1}\mathrm{s}^{-1}$.

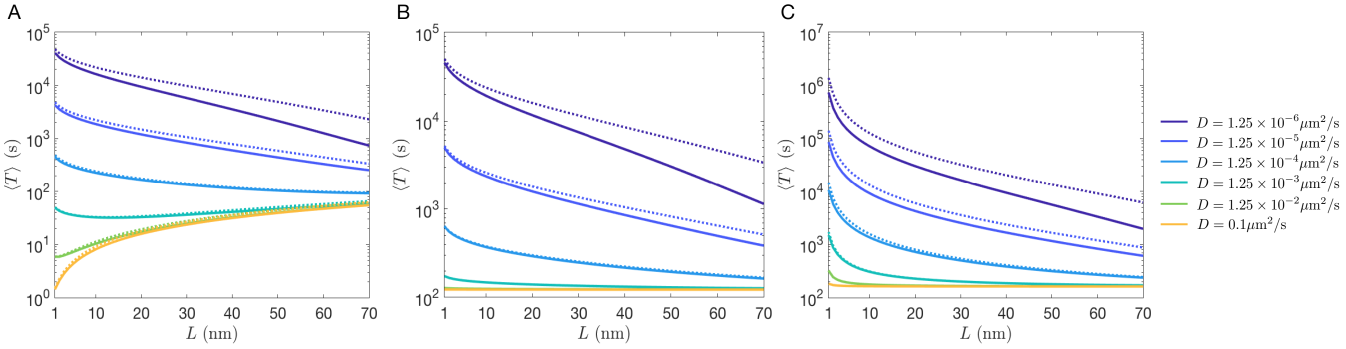

Figure S5: The well-mixed MRT $\langle T\rangle$ determined from the numerical solution of the Gaussian interaction model given by Eq. S15 (solid lines) and exact solution to the Doi step function interaction model given by Eq. 4 using calibrated λ and ε values (dashed lines). See discussion of SI Section S3. (A) 2.5D model, having Doi solution (Eq. 8) and calibration (Eq. 6); (B) 2D model, having Doi solution (Eq. 8) and calibration (Eq. S21); (C) 3D model, having Doi solution (Eq. S16) and calibration (Eq. 6). The area (2D)/volume (3D) of the circle/sphere is chosen to be the same as the square/cube of side length 300 nm. For A and C the catalytic rate k_{cat} is set to be 0.1 $\mu\text{M}^{-1}\text{s}^{-1}$. For B the 2D catalytic rate k_{cat} is $\frac{1}{3}\times 10^6$ $\mu\text{M}^{-1}\text{s}^{-1}\text{m}^{-1} = 553.4341$ $(\text{nm})^2\text{s}^{-1}$.

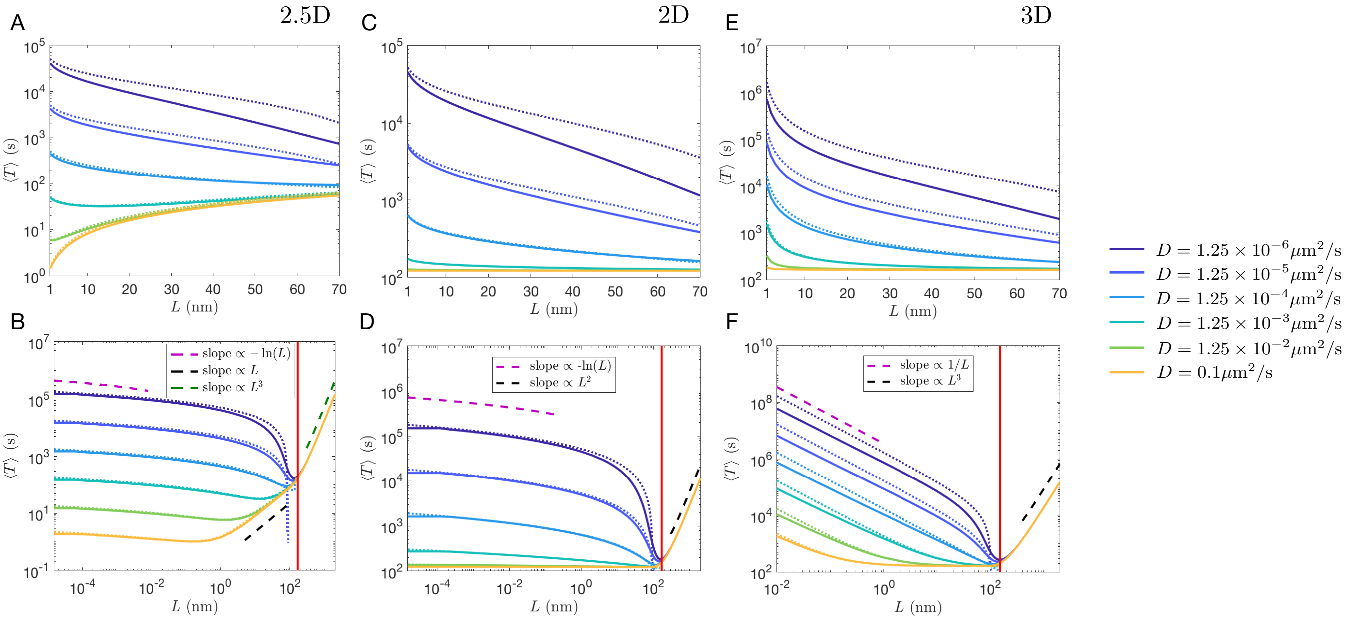

Figure S6: The well-mixed mean reaction time (MRT), $\langle T\rangle$, only demonstrates a switch in dependence on molecular reach for small vs. large diffusivities when considering membrane-bound molecules with cytosolic tails that react in 3D (2.5D model). In all figures solid lines correspond to $\langle T\rangle$ as estimated by numerically solving the ODE in Eq. S15. Dotted lines correspond to the asymptotic expansions in Eq. 11a for A/B, Eq. 11b for C/D and Eq. 11c for E/F. Dashed lines show general scaling behavior as a function of L. A) 2.5D model well-mixed MRT over physical parameter range. B) Same as A but showing an expanded range of L values. C) 2D model well-mixed MRT over physical parameter range. D) Same as C but showing an expanded range of L values. E) 3D model well-mixed MRT over physical parameter range. F) Same as E but showing an expanded range of L values. In the expanded range figures the red solid line gives the L value such that $\varepsilon/R = 1$, corresponding to when the Doi interaction distance, ε, is equal to the domain radius, R. Note, as $\varepsilon \to R$ from below the asymptotic expansions break down as $\varepsilon/R \not\ll 1$. For A, B, E, and F the catalytic rate k_{cat} is 0.1 $\mu\text{M}^{-1}\text{s}^{-1}$. For C and D the 2D catalytic rate k_{cat} is $\frac{1}{3}\times 10^6$ $\mu\text{M}^{-1}\text{s}^{-1}\text{m}^{-1} = 553.4341$ $(\text{nm})^2\text{s}^{-1}$.

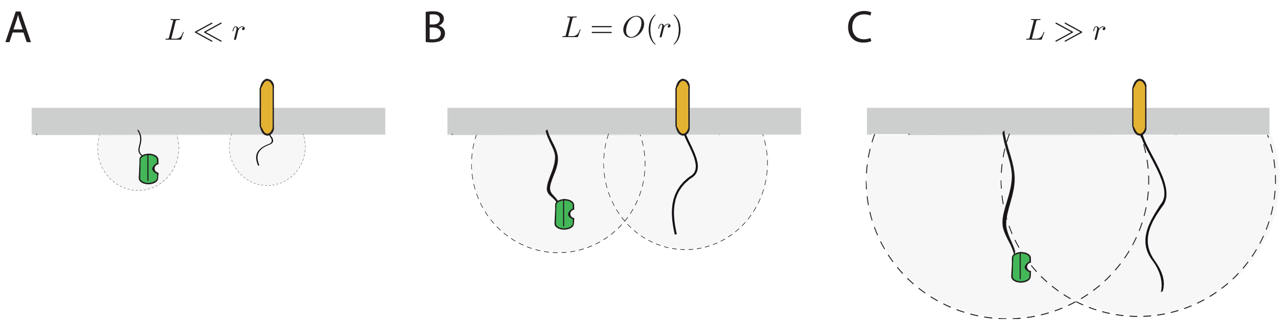

Figure S7: For *slowly* diffusing membrane-bound proteins, reaction potency is maximized when the reach, L, is comparable to the protein separation, r. **(A)** When the reach is much smaller than the protein separation, the cytosolic tails are too short to allow the reactive enzymatic/substrate sites to be in contact. **(B)** When the reach is comparable to the protein separation, the probability of contact between the reactive sites is maximized. **(C)** When the reach is much larger than the protein separation, the cytoplasmic tails explore too large a 3D volume proximal to the membrane so that the reactive sites rarely encounter each other.

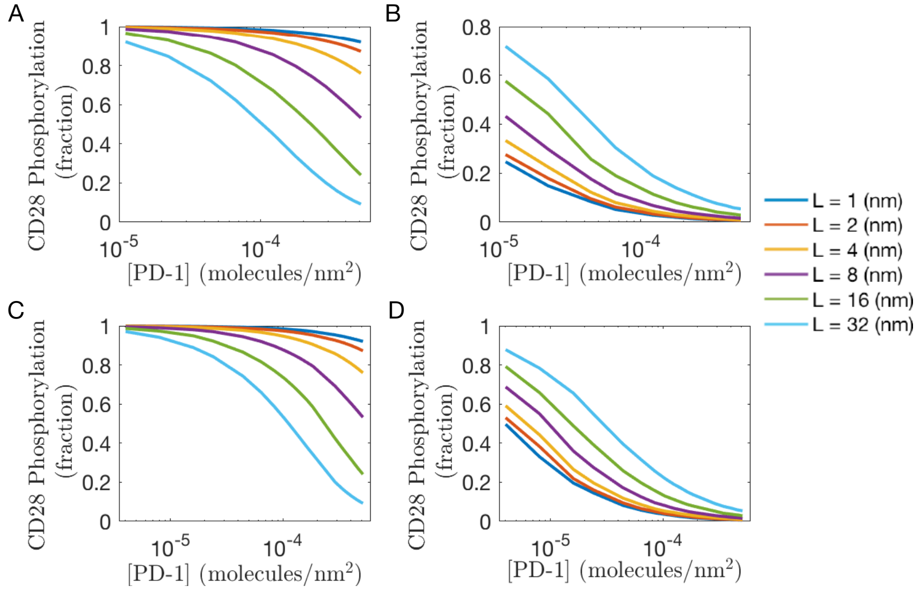

Figure S8: Dependence of the steady-state fraction of phosphorylated CD28 in the CD28 model for two different domain sizes when diffusion is slow (A,C; $D = 1.25 \times 10^{-6} \mu m^2/s$) and fast (B,D; $D = 1.25 \times 10^{-2} \mu m^2/s$). A) and B) The steady-state fraction of phosphorylated CD28 using a square domain of side length 300nm. C) and D) The steady-state fraction of phosphorylated CD28 using a square domain of side length 500nm. [PD-1] is shown on a logarithmic scale in each figure. Each curve was estimated from 100000 simulations. The catalytic rate k^*_{cat} was set to be $0.01 \mu \text{M}^{-1}\text{s}^{-1}$.

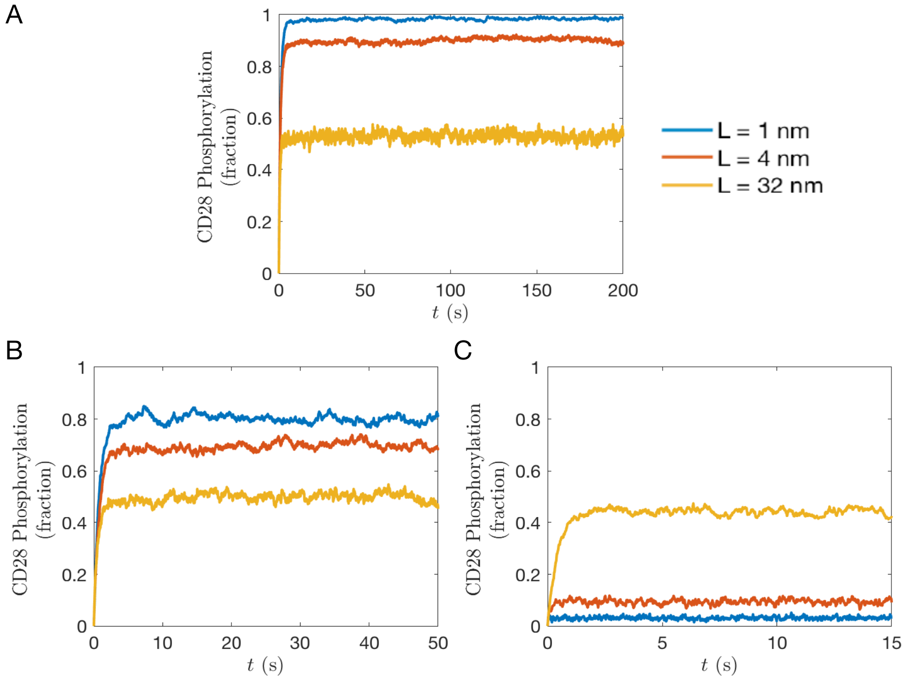

Figure S9: Average fractions of phosphorylated CD28 in the CD28 model versus time. Each figure ends at the termination time at which we concluded the system had reached steady-state. These times were then used in larger sets of simulations to produce Fig. 2. A) $D = 1.25 \times 10^{-6} \mu\mathrm{m}^2/s$. B) $D = 1.25 \times 10^{-4} \mu\mathrm{m}^2/s$. C) $D = 1.25 \times 10^{-1} \mu\mathrm{m}^2/s$. Each curve is an average from 100 simulations with an initial PD-1 concentration of $3.5556 \times 10^{-4}/\mathrm{nm}^2$. Remaining parameters are as in Table 1.

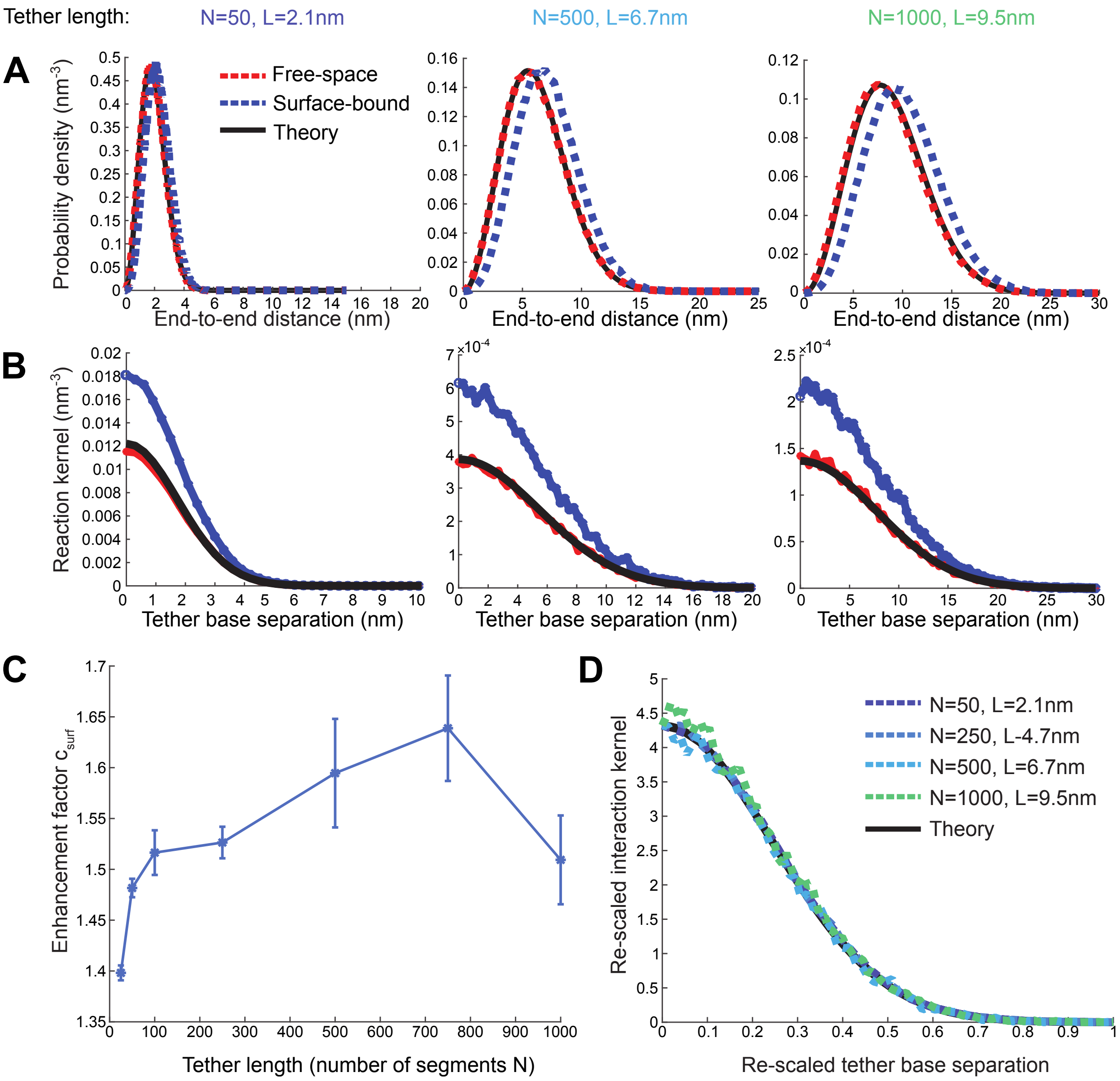

Figure S10: Simulation of tethers of various lengths adhered to a surface. (A) End-to-end distances for surface-bound tethers (blue dashed) have probability densities shifted slightly larger than free-space (red dashed). Free-space results can be compared with analytical theory (black solid) for validation. (B) Reaction kernel for surface-adhered tethers $\sigma_{\text{surf}}(r)$ (blue-dashed) and free-space $\sigma_{3\text{D}}(r)$ (red dashed). The surface enhances the reaction kernel by approximately 1.5-fold. Free-space result is compared to analytical theory (black solid). (C) Best-fit factor c_{surf} that fits the surface-adhered kernel (blue curves in (B)) to the approximation (Eq. S27). (D) If the reaction kernels from (B) are re-scaled according to Eq. S29, we find that all kernels, for the full range of tether length N we explored, collapse onto a single curve, demonstrating the validity of the approximation (Eq. S27) with this choice of enhancement factor c_{surf}.

Supporting References

References

1. Doi, M. (1976) Second quantization representation for classical many-particle system. *J. Phys. A: Math. Gen.* **9**, 1465–1477.

2. Doi, M. (1976) Stochastic theory of diffusion-controlled reaction. *J. Phys. A: Math. Gen.* **9**, 1479–1495.

3. Isaacson, S. A & Zhang, Y. (2018) An unstructured mesh convergent reaction-diffusion master equation for reversible reactions. *J. Comp. Phys.*

4. Isaacson, S. A. (2013) A convergent reaction-diffusion master equation. *J. Chem. Phys.* **139**, 054101–1 to 054101–12.

5. Gibson, M. A & Bruck, J. (2000) Efficient exact stochastic simulation of chemical systems with many species and many channels. *J. Phys. Chem. A* **104**, 1876–1899.

6. Gillespie, D. T. (1977) Exact stochastic simulation of coupled chemical-reactions. *J. Phys. Chem.* **81**, 2340–2361.

7. Bortz, A. B, Kalos, M. H, & Lebowitz, J. L. (1975) A new algorithm for Monte Carlo simulation of Ising spin systems. *J. Comp. Phys.* **17**, 10–18.

8. Bressloff, P. C. (2014) *Stochastic Processes in Cell Biology*, Interdisciplinary Applied Mathematics. (Springer) No. 41.

9. Cheviakov, A. F & Ward, M. J. (2011) Optimizing the principal eigenvalue of the Laplacian in a sphere with interior traps. *Mathematical and Computer Modelling* **53**, 1394–1409.

10. Szabo, A, Schulten, K, & Schulten, Z. (1980) First passage time approach to diffusion controlled reactions. *The Journal of Chemical Physics* **72**, 4350–4357.

11. Pastor, R. W, Zwanzig, R, & Szabo, A. (1996) Diffusion limited first contact of the ends of a polymer: Comparison of theory with simulation. *Journal of Chemical Physics* **105**, 3878–3882.

12. Sarkar, D, Brahmanandan, A, & Thakur, S. (2015) Dynamics of loop formation in active chains. *Macromolecular Symposia* **357**, 133–140.

13. Bryant, D, Clemens, L, & Allard, J. (2017) Computational simulation of formin-mediated actin polymerization predicts homologue-dependent mechanosensitivity. *Cytoskeleton* **74**.

14. Moglich, A, Joder, K, & Kiefhaber, T. (2006) End-to-end distance distributions and intrachain diffusion constants in unfolded polypeptide chains indicate intramolecular hydrogen bond formation. *Proceedings of the National Academy of Sciences* **103**, 12394–12399.

15. Krieger, F, Fierz, B, Bieri, O, Drewello, M, & Kiefhaber, T. (2003) Dynamics of unfolded polypeptide chains as model for the earliest steps in protein folding. *Journal of Molecular Biology* **332**, 265–274.

16. Lapidus, L. J, Eaton, W. A, & Hofrichter, J. (2000) Measuring the rate of intramolecular contact formation in polypeptides. *Proceedings of the National Academy of Sciences* **97**, 7220–7225.

17. Yeh, I. C & Hummer, G. (2003) Peptide dynamics from microsecond molecular dynamics simulations in explicit solvent. *Abstracts of Papers of the American Chemical Society* **226**, U424–U424.

18. Zhou, H.-X. (2001) Loops in Proteins Can Be Modeled as Worm-Like Chains. *The Journal of Physical Chemistry B* **105**, 6763–6766.

19. Lee, H, Venable, R. M, MacKerell Jr., A. D, & Pastor, R. W. (2008) Molecular Dynamics Studies of Polyethylene Oxide and Polyethylene Glycol: Hydrodynamic Radius and Shape Anisotropy. *Biophysj* **95**, 1590–1599.

20. Mukhopadhyay, H, de Wet, B, Clemens, L, Maini, P. K, Allard, J, Van Der Merwe, P. A, & Dushek, O. (2016) Multisite Phosphorylation Modulates the T Cell Receptor ζ-Chain Potency but not the Switchlike Response. *Biophysical Journal* **110**, 1896–1906.

21. Milner, S. T. (1991) Polymer brushes. *Science* **251**, 905–914.
